# Matching diagnostics development to clinical need: Target product profile development for a point of care test for community-acquired lower respiratory tract infection

**DOI:** 10.1371/journal.pone.0200531

**Published:** 2018-08-01

**Authors:** Micaela Gal, Nicholas A. Francis, Kerenza Hood, Jorge Villacian, Herman Goossens, Angela Watkins, Christopher C. Butler, the RAPP-ID consortium

**Affiliations:** 1 Division of Population Medicine, Medical School, Cardiff University, Cardiff, United Kingdom; 2 Centre for Trials Research, Cardiff University, Cardiff, United Kingdom; 3 Janssen Diagnostics BVBA, Beerse, Belgium; 4 Department of Clinical Pathology, University of Antwerp, Wilrijk, Belgium; 5 Nuffield Department of Primary Care Health Sciences, University of Oxford, Oxford, United Kingdom; University of California San Diego, UNITED STATES

## Abstract

**Background:**

Point of care tests (POCTs) are increasingly being promoted for guiding the primary medical care of community acquired lower respiratory tract infections (CA-LRTI). POCT development has seldom been guided by explicitly identified clinical need and requirements of the intended users. Approaches for identifying POCT priorities and developing target product profiles (TPPs) for POCTs in primary medical care are not well developed, and there is no published TPP for a CA-LRTI POCT aimed at developed countries.

**Methods:**

We conducted workshops with expert stakeholders and a survey with primary care clinicians to produce a target product profile (TPP) to guide the development of a clinically relevant and technologically feasible POCT for CA-LRTI.

**Results:**

Participants with clinical, academic, industrial, technological and basic scientific backgrounds contributed to four expert workshops, and 45 practicing primary care clinicians responded to an online survey and prioritised community-acquired pneumonia (CAP) as the CA-LRTI where a new POCT was most urgently needed. Consensus was reached on a TPP document that included information on the intended niche in the clinical pathway in primary medical care; diagnostic product specification (intended use statement and test concept), and minimum and ideal user specifications. Clinicians minimum requirements of a CA-LRTI POCT included the use of minimally invasive samples, a result in less than 30 minutes, no more than a single preparation step, minimum operational requirements, and detection of common respiratory pathogens and their resistance to commonly prescribed antibiotics.

**Conclusions:**

This multidisciplinary, multistage partnership approach generated a clinically-driven TPP for guiding the development of a new POCT, and this approach as well as the TPP itself may be useful to others developing a new POCT.

## Introduction

A primary care point of care test (POCT) is a test carried out near to the patient that generates a result without reference to a laboratory, and rapidly enough to affect patient management at the point of care [1, 2]. POCT development has often been driven by technological innovation rather than in response to a clearly defined unmet clinical need, and as a consequence many POCTS have not been taken up into routine clinical care. There are also some clinical niches where POCTs are urgently required. For example, there is a need for improved POCTs to help primary care clinicians to safely reduce and better target antibiotic prescribing for common infections such as community acquired lower respiratory tract infections (CA-LRTIs) [3], [4]. CA-LRTIs are one of the leading acute reasons for consulting in primary care, and 20%-95% of patients are prescribed an antibiotic [5], [6]. More than 80% of these antibiotics may be unnecessary as CA-LRTIs in developed countries are largely self-limiting and the majority of patients do not benefit meaningfully from antibiotic treatment [7], [8], [9]. A rapid POCT that is feasible for use in primary care consultations that helps clinicians decide when antibiotics can safely be withheld, or when antibiotic treatment is likely to benefit patients, could improve care of this common and important condition and help combat antibiotic resistance.

A critical factor in successful POCT development is gaining an in-depth understanding of the end users’ (e.g. clinicians) priorities, needs and operational requirements for any new POCT at the outset [10], [11]. This information, summarised in a target product profile (TPP), should guide test development so that it matches clinical need and safely improves outcomes in priority conditions. [12]. TPPs for diagnosing infections disease have been developed by the World Health Organisation (WHO), Foundation for Innovative New Diagnostics (FIND) and Médecines sans Frontiéres (MSF) [13] [14] [15] and others through the identification of stakeholders for consultation, priority setting to identify the highest priority for test development, defining operational and technical test characteristics, discussing disputed criteria, and obtaining final consensus [16, 17]. To formalise TTP’s and ensure their verification (consistency and completeness) formal modelling approaches have also been helpful [18] [19]. However, as yet, there is no consensus on the priority CA-LRTI where a POCT is most urgently needed, the ideal process for developing a POCT TPP, and there is no published TPP for a CA-LRTI for use in developed countries. Our experience of developing a TPP for the exemplar condition of CA-LRTI illustrates how a TPP can be generated to inform the development of a new POCT.

## Materials and methods

### Ethics

This study was reviewed and approved by the Cardiff University School of Medicine Ethics Committee (SMREC Reference Number 11/32).

### Study design

The development of our CA-LRTI TPP was an iterative process that included meetings with expert stakeholders from the EU Innovative Medicines Initiative (IMI) supported project, ‘*RAPP-ID*: *Rapid Point-of-Care Test for Infectious Diseases*’ (IMI RAPP-ID) project consortium (https://www.imi.europa.eu/content/rapp-id) and a web-based survey targeted at practicing primary care clinicians with an interest in CA-LRTI. Both qualitative and quantitative data informed the TPP.

### RAPP-ID consortium expert meetings and workshops

Four 2–3 day RAPP-ID stakeholder meetings, including breakout workshops targeted to the specific disease areas, were conducted between April 2011 and January 2012 (see Table 1 for meeting objectives). Stakeholders included expert and practicing clinicians, microbiologists, scientists (molecular microbiology, chemistry and physics), diagnostic market experts and test developers from academia and industry (European Federation of Pharmaceutical Industries and Associations (EFPIA)) in Europe. Learning points from the TRANS-Atlantic Task Force on Antimicrobial Resistance (TATFAR) meeting on *‘Challenges and solutions in the development of new diagnostic tests to combat antimicrobial resistance’* informed stakeholder discussions.

**Table 1:** Objectives of the stakeholder planning meetings.

No	Objective
**1**	Familiarise all stakeholders with the expertise and technologies available within the consortium
**2**	Discuss potential benefits of optimised disease diagnosis
**3**	Reach agreement on target conditions that would most benefit from improved diagnosis for adequate antimicrobial treatment
**4**	Consider the clinical complexity of antibiotic treatment decisions
**5**	Clarify the current state of the art in diagnostic tools for each disease condition
**6**	Reach agreement on micro-organisms, biomarkers and thresholds (e.g. limit of detection, colonisation vs. infection)
**7**	Discuss the availability and challenges of clinical sample availability, collection and processing
**8**	Develop models of therapeutic algorithms and patient stratification including POCT integration
**9**	Discuss potential scenarios from sample collection to read-out
**10**	Develop the user survey and discussing the technical feasibility of including the identified ideal user requirements

### User survey for CA-LRTI POCT

An on-line survey, informed by the stakeholder meeting outputs, was developed in collaboration with RAPP-ID consortium members to ensure questions covered information required by the test developers. The survey was piloted prior to distribution. Data collection was from 12th September to 12^th^ December 2011. The survey was aimed at practicing primary care clinicians with an interest in respiratory tract infections and was disseminated through RAPP-ID partners, the General Practice Respiratory Infections Network (GRIN), and flyers at the European Respiratory Society Annual meeting (Netherlands, September 2011).

**Fig 1:**
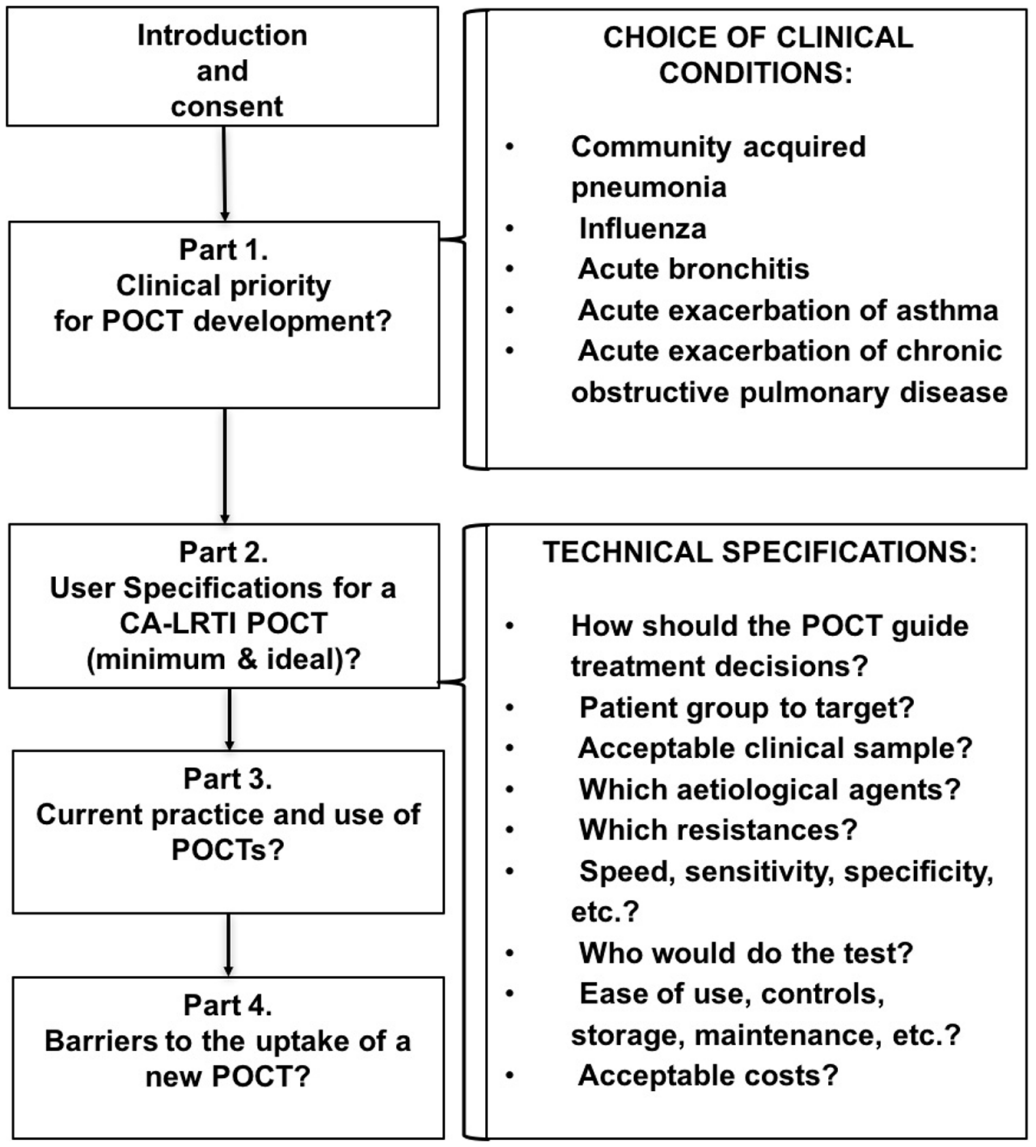
Survey to identify clinical need, minimum and ideal user requirements and perceived barriers for a new CA-LRTI POCT.

The survey had four parts (Fig 1). Part 1 aimed to identify the priority clinical LRTI sub-conditions that clinician’s considered would most benefit from improved rapid diagnostics. Clinical case definitions for each LRTI sub-type were included (S1 Table). Part 2 asked about clinician’s ideal and minimum user specifications for a CA-LRTI POCT. Parts 3 and 4 asked about clinician’s current POCT use and barriers to implementing POCT ‘s in routine care and microbiologists.

Questions to identify clinical priority or need for the POCT asked respondents to i) select their ideal requirement and ii) and assign a number of 1 to 5 (1-highest priority to 5-lowest priority) for their the requirement they considered most essential. To determine the POCT technical specifications, respondents were asked to i) select their ideal requirement and minimally acceptable requirement, and ii) select yes/no for questions asking if they would consider using a test with the listed characteristics. For all questions respondents could select more than one option and provide free text comments. Options for ‘unsure’ and ‘not relevant to my practice’ were included.

The results of the user survey were presented to all RAPP-ID partners and the technical feasibility of including the ideal user requirements into a POCT discussed with guidance from the expert clinicians

### Survey data analysis

Survey results were combined for all countries to inform the TPP. Questions asking respondents to select their ideal requirements and responses to binary (yes/no) questions were analysed as number and proportion as an average. For ranking questions, the majority of respondents selected and scored more than one priority for each question. These results were therefore also analysed as numbers and proportions as an average. Free text comments were included in the appendix of the TTP.

### Target product profile (TPP) document

Information from the stakeholder meetings and the user survey were used to design and inform the TPP (Table 2). Stakeholder discussions aimed to reach consensus on the final TPP including the specifications for a CA-LRTI POCT and a proof of concept POCT.

**Table 2:** Structure of the target product profile (TPP) document.

SECTIONS	CONTENTS
**Front of document**	Authorizations, appendix, glossary, definitions
**Aim of document**	Reference document for researchers, developers and manufacturers
**Source of document information**	Workshops, surveys, literature reviews
**Clinical need and requirement for new test**	Include clinical setting, fit into clinical pathway, POCT concept diagram, overview of current tests used in clinical practice
**Information on clinical samples**	Sample type, nature of samples and target molecules (e.g. biomarkers, pathogens), sampling equipment, acceptability of samples
**Diagnostic product specification**	Intended use statement, descriptions of test concept (description of the desired test) and proof of concept (test and methods that will demonstrate in principle the feasibility of developing a test with the desired TPP)
**TEST SPECIFICATIONS** **(Tables of key features including both (ideal (target) and minimum (acceptable) requirements)**.	
**Technical specifications**:	Intended use, clinical decision to be influenced, place of use, patient criteria, target molecule, performance against reference, type of analysis, readout system, sample type, reproducibility, compliance with regulators, reproducibility near clinical thresholds.
**Operational characteristics**:	Sample requirements (e.g. volume of sample, sample preparation, quality, requirement for precise volumes), controls, waste disposal, batching, end-user profile, training, biosafety, test speed, test stability, storage requirements, shelf life, lifetime of machine, need for additional equipment, cost.
**Market overview**	Cost of manufacturing single device, competitive landscape, regulatory pathway, competitive landscape, region of commercialization, market segmentation
**Information on Competitor Tests**	Available and those coming to market. Details on test performance, characteristics, market uptake etc.

## Results

### Clinician survey

#### Survey respondents

45 primary care clinicians completed the survey but not all respondents completed all survey questions. The majority of the respondents (21) were from the UK, and others from Belgium (9), The Netherlands (3), Poland (3), Spain (2), USA (2), Germany (1), Finland (1), Norway (1), Sweden (1) and Australia (1). 34 (70%) respondents were clinically research active, and their clinical workload relating to infections varied from <20% to 60%.

#### Clinical priority for POCT development

Community acquired pneumonia (CAP) was the LRTI given the highest overall ranking with 16 (35.6%) respondents giving it a ranking of 1 (high priority) and 34 (75.6%) giving a ranking of between 1 and 3. 11 (24.4%) respondents also selected influenza and acute exacerbations of chronic obstructive airways disease (aeCOPD) as their highest priority. Acute exacerbations of asthma and bronchitis were selected as the top priority by 7 (15.6%) and 4 (8.9%) respondents.

There were 88 free text responses (S2 Table).

#### Ideal and minimum CA-LRTI POCT requirements

For the majority of questions, respondents selected and ranked more than one option (Table 3).

**Table 3:** The gaps and needs for a priority CA-LRTI POCT as defined by an expert consensus process with primary care clinicians.

Question (number of respondents completing question on ideal and minimum specification)	Answer Options	Ideal specification (number (%) selecting as top priority)	Minimum specification (number (%) selecting as top priority)
**How would a new CA-LRTI POCT best guide treatment decisions?** (41)	Treatment monitoring; Initial treatment targeting (prescribing optimal and appropriate antibiotic) De-escalation of treatment from earlier more powerful (broad-spectrum) to less powerful appropriate antibiotic	Initial treatment targeting (40, (97.6))	Initial treatment targeting (34 (82.9))
**What patient sub-group should the test benefit?** (38)	Neonates; Children; Adults <65 years; Adults 65–80 years; Adults >80 years	Adults aged 65–80 years (25 (65.8))	Adults aged 65–80 years (19 (48.7))
**Antibiotic status of patients for testing?** (41)	Antibiotic pre-treated; Antibiotic naïve (no previous antibiotic treatment for that episode) All patients	All patients including antibiotic pre-treated and antibiotic naïve patients (17 (41.5))	Antibiotic naïve (25 (65.8))
**What category of staff would use the test most often?** (40)	Doctors Nurses Practice nurse Nurse practitioner	Doctors (33 (82.5))	Doctors (29 (70.7))
**What aetiological agents do you think are most important for the test to detect?** (42)	Bacteria Viruses	Bacteria (36 (85.7))	Not Available (NA)
**Which do you think are the most important bacteria for the test to detect?** (35)	List of common respiratory pathogens Other Unsure	*S*. *pneumoniae* (26 (74.3)); *H*. *influenzae* (21 (60.0)); *M*. *pneumoniae* (17 (48.6)); Unsure (8 (22.9)).	NA
**What level of bacterial identification is required?** (34 and 19)	Gram-positive / Gram-negative Genus level (e.g. *Streptococcus* spp) Species level (e.g. *S*. *pneumoniae*) Genus level and quantification Species level and quantification Unsure 4&5) and e.g. for de-escalation of treatment or colonisation vs. infection	Gram-positive / Gram-negative (9 (26.5)); Species level (8 (23.5)); Unsure (13 (38)).	Gram-positive / Gram-negative (8(50))
**Would information about antibiotic resistance change your clinical practice?** (40)	Yes No	Yes (39 (97.5))	NA
**What resistance do you think the test should detect?** (36)	List of antibiotics (including generic names and groups); Other	Penicillin’s (25 (69.4)); Macrolides (17 (47.2)); Amoxicillin-Clavulanate (16 (44.4)); Beta-lactamases (14 (38.9)); Fluoroquinolones (14 (38.9)); Cephalosporins (13 (36.1))	NA
**Level of antibiotic susceptibility information required?** (38 and 21)	Resistance gene absent; Resistance gene present; Sensitive/resistant corresponding to antibiotic breakpoint; Unsure	Sensitive/resistant corresponding to antibiotic breakpoints (24 (63.2)); Unsure (13 (34))	Sensitive/resistant corresponding to antibiotic breakpoints (17 (81))
**Which do you think are the most important viruses for the test to detect?** (11)	List of common respiratory viruses; Other; Unsure	Influenza (10 (90.9)); Respiratory syncytial virus (6 (54.5)); Para-influenza and adenoviruses (4 (36.4))	NA
**Level of viral identification required?** (12 and 9)	Species identification; Species and strain identification; Species and viral load; Flu A/B discrimination; Unsure	Species level identification (6 (50)); Unsure (4 (33.3))	Species level identification (5 (55.6))
**Would information about antiviral resistance change your clinical practice?** (39)	Yes; No	Yes 25 (64.1))	NA
**Most important antiviral resistance to detect?** (14 and 8)	List of antivirals; Other; Unsure	Oseltamivir (Tamiflu) (8 (57.1)); Unsure (4(28.6))	Oseltamivir (7 987.5))
**Which test prediction is important to you?** (36 and 30)	Rate of true positives Rate of true negatives Rate of false positives Rate of false negatives Unsure	Rate of true positives (20 (55.6)); Rate of true negatives (16 (44.4)); Unsure (5 (13.9))	Rate of true negatives (18 (62.1))
**Total time taken from taking patient sample to receiving test result?** (35 and 31)	<30 minutes <1 hour 1–2 hours 2–4 hors >6 hours Other	<30 minutes (30 (85.7))	<30 minutes (22 (81.5))

#### Technical and operational requirement specifications of the POCT

Questions asked respondents to i) select their ideal requirements for POCTs, and ii) select the specifications that would prevent them considering a POCT (Table 4), and were invited to provide free-text comments (S3 Table).

**Table 4:** Clinicians’ technical and operational requirements, and specifications preventing test use.

TECNHICAL/OPERATIONAL FEATURE	CLINICIANS IDEAL REQUIREMENTS (number of respondents selecting as top priority (percentage))	SPECIFICATION THAT WOULD PREVENT CONSIDERATION OF A POCT (number of respondents (percentage))
**Acceptable clinical samples**	Throat swabs (28 (77.8)); Urine, nasal swabs, sputum, capillary blood and exhaled breath (20 (>55)); 31 (88.6) of respondents would consider a test that required a breath sample	Faecal samples (31 (88.66)) Induced sputum (27 (84.4)) Venous blood (11 (31.4))
**Sample preparation procedures**	The ability to use approximate volumes and a single preparation step (26 (74.3)); Not prone to contamination and no safety containment issues (25 (75.3) and 21 (61.8))	Requires >3 preparation steps (27 (84.4)); Requires >2 preparation steps (19 (59.4); Highly sensitive to contamination (22 (66.7))
**Storage of test and reagents**	Stability at room temperature (36 (100)); Minimum shelf life of 12 months (23 (63.9))	Shelf life of less than 6 months (17 (50)); Unstable at room temperature (11 (32.4));
**Test kit requirements**	Totally self-contained kit (30 (85.7)); No calibration required (20 (57.1)); Needs to contain the specimen collection device (15 (42.9))	Requires calibration before each test (19 (59.4)); Does not contain the specimen collection device (15 (45.5)); Kit not totally self-contained (13 (37.1))
**Control requirements**	All controls must be included as part of the kit (26 (74.3)); No need for external quality control (22 (62.9)); Needs to include controls within the kit (12 (34.3));	External quality control needed 18 (54.5)); Kit does not include controls (18 (52.9)); Kit does not include controls as part of each test (15 (48.4))
**Instrumentation requirements**	The instrument must be robust (24 (66.7)); No maintenance required, fits into a small area, and hand-held (> 21(61.8%)) for each specification)	Requires separate containment area (27 (77.1%)); Requires monthly maintenance (24 (68.6%)); Fragile (22 (62.9))
**Test result requirements**	Easy to read (31 (86.1)); Connectivity enabling downloading of results to patient records (25 (69.4)); Unambiguous results, a simple yes/no/invalid readout and readable for at least an hour (>20 61.8%) for each specification)	Ambiguous results (28 (82.4)); Complex to read (22 (62.9)); Does not allow automatic download to patient record (4 (11.8)).
**Acceptable training requirements?**	Test can be performed by any healthcare worker without training (24 (70.6); Training time of one day maximum (22 (64.7)); Self-administration by patients (11 (32.4))	Requires more than one day training (25 (71.4)); Can only be performed by an experience healthcare worker (11 (31.4))
**Power requirement**	Powered by battery (30 (88.2)); Standard mains (27 (79.4))	Could not be powered by standard mains (7 (20.6)); Could not be powered by battery (4 (11.8))
**Acceptable maximum cost per patient test**	No more than €10 (25 (73.5)); No more than €20 (11 (32.4)	NA
**Acceptable maximum instrumentation cost**	<€1,000 (20 (58.8)); €5,000 (9 (26.5)); No cost (14 (41.2))	NA

#### Current use of POCTs

21/36 of respondents (58.3%) did not use any POCT for managing respiratory infections, 7 (19.4%) used a CRP test and five (13.9%) a ‘Strep A’ test. When suspecting a false positive POCT result, 22 (64.7%) respondents would follow-up the patient, and 16 (47.1%) would re-test; 21 (63.6%) would follow-up the patient; 16 (48.5%) would re-test; 11 (33.3%) would use clinical algorithms, and; 10 (27.8%) would refer samples to a laboratory or conduct additional tests. In relation to a question on laboratory results, 8 (24.2%) respondents indicated that they would ignore laboratory results if they suspected a false negative and 7 (21.2%) would prescribe a broad-spectrum antibiotic.

#### Clinician’s perceived barriers to the uptake of a new POCT

29 respondents provided free-text comments (S4 Table). Speed and cost of the test were most frequently perceived barrier (n = 19 (65.5%)). Other barriers related to test cost versus clinical benefit (n = 5 (17.3%)); test complexity and training (n = 19 (65.5%)); time and workload (n = 10 (34.5%)); performance, accuracy and reliability (n = 7 (24.1%)), and patient acceptability (n = 3 (10.3%)).

#### The target product profile (TPP) document

Consensus on a final TPP to guide the CA-LRTI POCT development included the primary care clinician’s top priority and ideal and their minimum test performance requirements. The TPP document also included the POCT diagnostic product specification statement, a description of the test concept and a proposed proof of concept evaluation (Table 5). The test concept provides a detailed description of the desired POCT. The proof of concept test describes the test and methods to demonstrate, in principle, the feasibility of developing a POCT that matches the TPP. Details of the clinical pathway were also included to promote understanding of the test setting for all the project stakeholders. Examples of currently available tests and emerging technologies, the current market and regulatory pathway for POCT development were also reviewed and included as summary information in the TPP document. The TPP document is available as supplementary material (S1 File)

**Table 5:** The diagnostic product specification statement.

**INTENDED USE(S) OF THE TEST**:
A POCT to enhance the initial clinical management of suspected CAP in adult patients presenting in primary care. The POCT should achieve this by indicating with sufficient precision the presence or absence of the organisms that account for almost all cases of CAP, so as to inform decisions about whether an immediate antibiotic prescription is necessary or not. The primary target population is in primary care. The POCT should be easy to use by a range of health care professionals in a variety of settings (e.g. doctors, nurses and any community health workers in any primary care setting doctors surgery, GP home visit, and nursing homes). The POCT should be feasible and cost effective in the primary care setting. The test is designed to be used with either nasopharyngeal (NP) swab or exhaled breath samples without need for prior culture.
**TEST CONCEPT**:
The POCT should be able to detect *Streptococcus pneumoniae*, *Haemophilus influenzae*, *Staphylococcus aureus*, *Moraxella catharralis*, *Chlamydophila pneumoniae*, *Mycoplasma pneumoniae*, *Legionella* spp and common viral respiratory pathogens including rhinovirus, influenza A and B, coronaviruses, human metapneumoviruses, respiratory syncytial virus (RSV), and parainfluenza. The POCT should be able to detect penicillin and macrolide resistance in common potentially pathogenic respiratory bacteria. The POCT should provide a technologically flexible platform that allows the future incorporation of biomarker detection, should biomarkers be identified that will add to diagnostic performance.
**PROOF OF CONCEPT TEST**:
A POCT to identify S. pneumoniae, H. influenzae, S. aureus, M. catharralis, C. pneumoniae, M. pneumoniae, Legionella spp and common viral respiratory pathogens including rhinovirus, influenza A and B, coronaviruses, human metapneumoviruses, respiratory syncytial virus (RSV), and para-influenza. The results of the POCT should be used to accurately identify the presence of common pathogenic respiratory bacteria and viruses in adult patients presenting with symptoms of CA-LRTI, to rapidly guide clinicians in their decisions whether or not to prescribe antibiotic or antiviral treatment.

## Discussion

We have described an approach for ensuring that that the development of a new diagnostic is driven by clinical need and the requirements of the clinical end users. Critical steps to identify gaps and needs for a new POCT included multidisciplinary stakeholder meetings that generated critically useful information and informed a survey of clinicians, both of which identified clinical priorities and the minimum and ideal performance characteristics of a new POCT.

Nine months was allocated for the initial planning stage of the RAPP-ID project, including the development of the TPP. This included workshops to ensure a shared understanding and vision was reached between multidisciplinary stakeholders. Scientists and technologists were introduced to the clinical need, the nature of the clinical setting, the clinical samples that could be obtained, the potential pathogens and challenges of establishing minimally acceptable performance thresholds. Clinicians gained an understanding of the available broad approaches, technologies and manufacturing restrictions. Involving all stakeholders in the design of the clinician survey and the TPP ensured that all information required by the technologists, test developers and manufacturers was captured.

Community acquired pneumonia was the clinical priority for POCT development. Clinicians minimum requirements for a CA-LRTI POCT included; that the test can be performed by doctors, uses minimally invasive samples, gives a result that is easy to interpret in less than 30 minutes, requires no more than a single preparation step, has minimum operational requirements, and can detect common respiratory pathogens and resistance to antibiotics commonly prescribed in primary care.

### Comparison with existing literature

Previous studies have described methods for consulting target users in the early development of new diagnostics, and have identified product failures arising from a disconnect between technology developers and the end users [11], [20] [21], [22]. Perceived barriers to involving actual users in the design and development process include lack of time and requirements for ethical approval [23].

Projects to develop new POCTs are complex and require multi-disciplinary collaborations with partners that have a wide range of expertise [24].

A number of TPPs have been developed for managing infectious diseases in resource-limited settings and these have focussed on detection of the infecting agent or differentiating between bacterial and non-bacterial infections to reduce antimicrobial overuse [13, 14, 17, 25, 26]. Clinicians’ views of POCTs for common infections including LRTI have been established in other studies [10], [27], [28], [29]. However, we were not able to identify any published TPPs for a primary care CA-LRTI POCT in developed countries or studies that identify which particular CA-LRTI condition would most benefit from a new POCT.

While our study identified many positive reasons for developing a POCT for CA-LRTI, some barriers were also highlighted. These included that a test focussing on aetiology may lead to an antibiotic prescription for all bacterial infections, including those that are self-limiting, and that in many cases, diagnosis and prognosis was obvious on clinical grounds alone.

There may be numerous current technical barriers to achieving a test to meet many of the clinicians ‘ideal’ requirements. The ideal specifications also need to be considered in light of the risks that they may pose to successful POCT development.

#### Strengths and limitations

The clinician survey was relatively small and the sample was largely one of convenience. However, respondents were practicing primary care clinicians with an interest in CA-LRTI. The survey instrument was relatively long which may led to incomplete responses. The TPP was confidential and dissemination was initially limited to the RAPP-ID consortium until the end of the project, which resulted in a delay to placing it in the public domain. Ideally, a formal validation of the TPP would also have been conducted. To investigate interesting relations between variables and add depth to the data analysis additional methods such as association rule learning and data mining technology could have been used.

## Conclusion

The development and consensus for a TPP ensures cross-disciplinary dialogue and shared understanding and focuses technological innovation on meeting prospectively identified clinical need. Looking ahead, a TPP should be regularly reviewed throughout product development to take into the account any changing clinical priorities, emerging technologies, and barriers that were unforeseen during development and evaluation stages [30].

## Supporting information

S1 Table Definitions of lower respiratory tract infections provided in the survey. (PDF)

S2 Table Comments on clinicians’ priority for the development of a new POCT. (PDF)

S3 Table Comments received in response to technical and operational survey questions. (PDF)

S4 Table Clinicians perceived barriers to the uptake of a new POCT in primary care. (PDF)

S1 File CA-LRTI POCT target product profile document. (PDF)

## References

[1] Hobbs R. Near patient testing in primary care. BMJ. 1996;312(7026):263–4. . 8611768 10.1136/bmj.312.7026.263 PMC2349898

[2] Delaney BC, Hyde CJ, McManus RJ, Wilson S, Fitzmaurice DA, Jowett S, et al Systematic review of near patient test evaluations in primary care. BMJ. 1999;319(7213):824–7. . 10496828 10.1136/bmj.319.7213.824 PMC314212

[3] St John A, Price CP. Existing and Emerging Technologies for Point-of-Care Testing. Clin Biochem Rev. 2014;35(3):155–67. . 25336761 PMC4204237

[4] Cooke J, Butler C, Hopstaken R, Dryden MS, McNulty C, Hurding S, et al Narrative review of primary care point-of-care testing (POCT) and antibacterial use in respiratory tract infection (RTI). BMJ Open Respir Res. 2015;2(1):e000086 10.1136/bmjresp-2015-000086 . 25973210 PMC4426285

[5] Macfarlane J, Holmes W, Gard P, Macfarlane R, Rose D, Weston V, et al Prospective study of the incidence, aetiology and outcome of adult lower respiratory tract illness in the community. Thorax. 2001;56(2):109–14. 10.1136/thorax.56.2.109 . 11209098 PMC1746009

[6] Woodhead M, Blasi F, Ewig S, Huchon G, Ieven M, Ortqvist A, et al Guidelines for the management of adult lower respiratory tract infections. Eur Respir J. 2005;26(6):1138–80. 10.1183/09031936.05.00055705 . 16319346

[7] Gonzales R, Steiner JF, Sande MA. Antibiotic prescribing for adults with colds, upper respiratory tract infections, and bronchitis by ambulatory care physicians. JAMA. 1997;278(11):901–4. . 9302241

[8] Kuyvenhoven MM, Verheij TJ, de Melker RA, van der Velden J. Antimicrobial agents in lower respiratory tract infections in Dutch general practice. Br J Gen Pract. 2000;50(451):133–4. . 10750213 PMC1313633

[9] Macfarlane J, Lewis SA, Macfarlane R, Holmes W. Contemporary use of antibiotics in 1089 adults presenting with acute lower respiratory tract illness in general practice in the U.K.: implications for developing management guidelines. Respir Med. 1997;91(7):427–34. . 9327045 10.1016/s0954-6111(97)90258-4

[10] Butler CC, Simpson S, Wood F. General practitioners' perceptions of introducing near-patient testing for common infections into routine primary care: a qualitative study. Scand J Prim Health Care. 2008;26(1):17–21. 10.1080/02813430701726285 . 18297558 PMC3406622

[11] Weigl BH, Gaydos CA, Kost G, Beyette FR Jr., Sabourin S, Rompalo A, et al The Value of Clinical Needs Assessments for Point-of-Care Diagnostics. Point Care. 2012;11(2):108–13. 10.1097/POC.0b013e31825a241e . 23935405 PMC3737000

[12] Shah SG, Robinson I. Benefits of and barriers to involving users in medical device technology development and evaluation. Int J Technol Assess Health Care. 2007;23(1):131–7. 10.1017/S0266462307051677 . 17234027

[13] FIND. Target Product Profiles [cited 2017 6 Nov]. https://www.finddx.org/target-product-profiles/.

[14] WHO. High-priority target product profiles for new tuberculosis diagnostics:report of a consensus meeting 2014 [cited 2017 6 November]. http://www.who.int/tb/publications/tpp_report/en/.

[15] MSF. Defining Specifications for a TB Point-of-Care Test 2009 [cited 2017 6 Nov]. https://www.msfaccess.org/sites/default/files/MSF_assets/TB/Docs/TB_event_POC_meetingoutcomes_full_ENG_2008.pdf.

[16] Dittrich S, Tadesse BT, Moussy F, Chua A, Zorzet A, Tangden T, et al Target Product Profile for a Diagnostic Assay to Differentiate between Bacterial and Non-Bacterial Infections and Reduce Antimicrobial Overuse in Resource-Limited Settings: An Expert Consensus. Plos One. 2016;11(8). 10.1371/journal.pone.0161721 PMC4999186 27559728

[17] Reipold EI, Easterbrook P, Trianni A, Panneer N, Krakower D, Ongarello S, et al Optimising diagnosis of viraemic hepatitis C infection: the development of a target product profile. Bmc Infectious Diseases. 2017;17. 10.1186/s12879-017-2770-5 PMC5688443 29143620

[18] Kamsu-Foguem B, Chapurlat V. Requirements modelling and formal analysis using graph operations. Int J Prod Res. 2006;44(17):3451–70. 10.1080/00207540500499377

[19] Kamsu-Foguem B, Tchuente-Foguem G, Foguem C. Conceptual graph operations for formal visual reasoning in the medical domain. Irbm. 2014;35(5):262–70. 10.1016/j.irbm.2014.04.001

[20] Martin JL, Murphy E, Crowe JA, Norris BJ. Capturing user requirements in medical device development: the role of ergonomics. Physiol Meas. 2006;27(8):R49–62. 10.1088/0967-3334/27/8/R01 . 16772664

[21] Drain PK, Hyle EP, Noubary F, Freedberg KA, Wilson D, Bishai WR, et al Diagnostic point-of-care tests in resource-limited settings. Lancet Infect Dis. 2014;14(3):239–49. 10.1016/S1473-3099(13)70250-0 . 24332389 PMC4016042

[22] Lemaire JF, Casenghi M. New diagnostics for tuberculosis: fulfilling patient needs first. J Int AIDS Soc. 2010;13:40 10.1186/1758-2652-13-40 . 20973946 PMC2987791

[23] Money AG, Barnett J, Kuljis J, Craven MP, Martin JL, Young T. The role of the user within the medical device design and development process: medical device manufacturers' perspectives. BMC Med Inform Decis Mak. 2011;11:15 10.1186/1472-6947-11-15 . 21356097 PMC3058010

[24] Syed SN, Ducrotoy MJ, Bachmann TT. Antimicrobial resistance diagnostics: time to call in the young? Lancet Infect Dis. 2016;16(5):519–21. 10.1016/S1473-3099(16)30011-1 . 27599645

[25] Organisation WH. High-priority target product profiles for new tuberculosis diagnostics:report of a consensus meeting 2014 [cited 2017 13 Nov]. http://www.who.int/tb/publications/tpp_report/en/.

[26] Dittrich S, Tadesse BT, Moussy F, Chua A, Zorzet A, Tangden T, et al Target Product Profile for a Diagnostic Assay to Differentiate between Bacterial and Non-Bacterial Infections and Reduce Antimicrobial Overuse in Resource-Limited Settings: An Expert Consensus. PLoS One. 2016;11(8):e0161721 10.1371/journal.pone.0161721 . 27559728 PMC4999186

[27] Wood F, Brookes-Howell L, Hood K, Cooper L, Verheij T, Goossens H, et al A multi-country qualitative study of clinicians' and patients' views on point of care tests for lower respiratory tract infection. Fam Pract. 2011;28(6):661–9. 10.1093/fampra/cmr031 . 21653924

[28] Turner PJ, Van den Bruel A, Jones CH, Pluddemann A, Heneghan C, Thompson MJ, et al Point-of-care testing in UK primary care: a survey to establish clinical needs. Fam Pract. 2016;33(4):388–94. 10.1093/fampra/cmw018 . 27048525 PMC4957010

[29] Howick J, Cals JW, Jones C, Price CP, Pluddemann A, Heneghan C, et al Current and future use of point-of-care tests in primary care: an international survey in Australia, Belgium, The Netherlands, the UK and the USA. BMJ Open. 2014;4(8):e005611 10.1136/bmjopen-2014-005611 . 25107438 PMC4127935

[30] Kamsu-Foguem B, Tiako PF, Mutafungwa E, Foguem C. Knowledge-based modelling applied to synucleinopathies. European Geriatric Medicine. 2015;6(4):381–8.

