# Supplementary material for: Matching diagnostics development to clinical need: Target product profile development for a point of care test for community-acquired lower respiratory tract infection
Source: PLoS One. 2018 Aug 1;13(8):e0200531. doi: 10.1371/journal.pone.0200531 (PMC6070214; doi:10.1371/journal.pone.0200531)
Supplement: S1 Table — (PDF) [file pone.0200531.s001.pdf]

S1 Table. Definitions of lower respiratory tract infections provided in the questionnaire

CLINICAL CONDITION	CLINICAL DEFINITION
**Community acquired pneumonia (CAP)**	An acute infection of the pulmonary parenchyma that is associated with at least some symptoms of acute infection accompanied by ausculatory findings consistent with pneumonia (such as localized breath sounds and localized rales) and/or the presence of an acute infiltrate on chest radiograph, in a patient not hospitalized or residing in a long-term care facility for 14 days prior to onset of symptoms.
**Influenza**	A person presenting with influenza like illness. Including both seasonal and pandemic influenza.
**Acute exacerbation of asthma**	The acute onset of an increase in asthma symptoms (e.g. dyspnoea, wheeze), a productive cough, and increase and/or change in colour (e.g. green or yellow) of sputum and/or other symptoms suggestive of infection.
**Acute exacerbation of chronic obstructive pulmonary disease (aeCOPD)**	A combination of, or all three of; worsening of dyspnoea, increase in sputum purulence and volume, as well as one of the following clinical criteria: an upper respiratory infection in the past five days; fever without another apparent cause; increased wheezing; increased cough; or increase in respiratory rate or heart rate by 20% above the baseline.
**Acute bronchitis**	The acute or sub-acute onset of cough accompanied with evidence of sputum production in a patient with no history of chronic pulmonary disease and no evidence of pneumonia or sinusitis.
