# Supplementary material for: Matching diagnostics development to clinical need: Target product profile development for a point of care test for community-acquired lower respiratory tract infection
Source: PLoS One. 2018 Aug 1;13(8):e0200531. doi: 10.1371/journal.pone.0200531 (PMC6070214; doi:10.1371/journal.pone.0200531)
Supplement: S2 Table — (PDF) [file pone.0200531.s002.pdf]

S2 Table. Comments on clinicians' priority for the development of a new POCT

CLINICAL CONDITION	NUMBER OF POSITIVE RESPONSES (N) AND EXAMPLES OF COMMENTS	NUMBER OF NEGATIVE RESPONSES (N) AND EXAMPLES OF COMMENTS
**Community acquired pneumonia (CAP)**	(11)<br><br>*'Differentiating serious pulmonary infection (pneumonia) from other respiratory, mostly self-limiting RTIs is important for management and prognosis. Clinical diagnosis of pneumonia is poor!'*	(9)<br><br>*'In this case there are sufficient diagnostic signs to base a decision on'*<br><br>*'I would treat with antibiotics anyway'*
***Influenza**	*(8)*<br><br>*'Identification of influenza would be useful both from epidemiological point of view and for targeting treatment and advice of the patient'*<br><br>*'In a busy flu season it would be helpful to identify patients who might require antibiotic for another pathogen or added infection'*<br><br>*'Influenza is often mixed with influenza-like illness; would be great if we could more objectively separate influenza from other LRTI, as well as following-up the patient during illness period; influenza is also frequently found in pneumonia patients'*<br><br>*'..need diagnostic help for pandemic influenza. Diagnostics for seasonal influenza would be a profoundly inappropriate use of resources'*	*(8)*<br><br>*'It would not change my management. Antivirals are usually not indicated. Maybe there could be some use in certain risk patients'*<br><br>*'I see no reason to do extra testing in such patient without clinical suspicions of complications'*
**Acute exacerbation of asthma**	*(7)*<br><br>*'The decision as to whether or not to treat possible associated LRTI in exacerbations of asthma is a recurrent issue in GP. To be able to distinguish between LRTI and viral urticaria would be of great help'*<br><br>*'The risk of further complication from asthma exacerbation could be decreased if we can totally rule out a bacterial aetiology'*	*(5)*<br><br>*'The treatment of exacerbation of asthma depends on the degree of dyspnea. A POC spirometry could be very useful. I don't know if a POC to detect infection would be useful'*<br><br>*'A negative test could not change my management'*
**Acute exacerbation of chronic obstructive pulmonary disease (aeCOPD)**	(8)<br><br>*'A POCT could avoid overprescribing antibiotics'*<br><br>*'Useful especially in chronic patients to know when antibiotics might be useful'*<br><br>*'Recurrent antibiotic treatments that may increase the risk of the patient to harbour resistant bacteria. A test that would help target the treatment would be useful'*	*(3)*<br><br>*'This is pretty much a clinical diagnosis; can't see much benefit in POC for this presentation'*<br><br>*'Would still treat with antibiotics plus steroids as per local protocols'*

<table>
<tr><td>Acute bronchitis</td><td>(5)

‘Important to differentiate acute bronchitis from pneumonia; clinically hard to differentiate in primary care, but acute bronchitis rarely asks for antibiotic management. However, up to 80% of acute bronchitis will be treated with pneumonia’

‘A negative test would support me in the management’</td><td>(9)

‘Clinical history and a thorough auscultation can be in most of the cases enough to make the diagnosis’

‘Most of these are pretty straightforward; only need test to distinguish from pneumonia in some cases’</td></tr>
<tr><td>Additional clinical priorities for development of a new POCT</td><td colspan="2">(4)

‘It may be hard to distinguish bronchitis from atypical pneumonia (Chlamydia p., Mycoplasma p.).

It would be very useful with a POCT to single out atypical pneumonia’

It would be helpful to have a POCT for specific potentially serious infections like Legionella (already existing), Coxiella burnettii infections… SARS, etc.

(6)

A test that allows differentiation between CAP and bronchitis would be useful.</td></tr>
<tr><td>Additional comments on POCT development</td><td colspan="2">(5)

‘POCT potentially useful and could save patients a trip to hospital;

‘I think that the place of POCT is at the end of clinical reasoning where uncertainty regarding treatment remains. Treating the results instead of the clinical diagnosis and picture will shift and not diminish the use of antibiotics’

‘ Apart from the scientific arguments to consider POCT for daily general practice other arguments are becoming more relevant: fear of missing serious illnesses, less (societal) acceptance of false-negative results, despite of enlarging risk of false positive results, service for patients, (double) aging problem, etc’

‘A pitfall could be that every POCT positive for bacterie leads to an antibiotic prescription’

‘In general, the use of near patient testing would be less useful when a patient has ‘barn door’ symptoms of illness as it’s unlikely that my management would change significantly’</td></tr>
</table>
