# Supplementary material for: Matching diagnostics development to clinical need: Target product profile development for a point of care test for community-acquired lower respiratory tract infection
Source: PLoS One. 2018 Aug 1;13(8):e0200531. doi: 10.1371/journal.pone.0200531 (PMC6070214; doi:10.1371/journal.pone.0200531)
Supplement: S3 Table — (PDF) [file pone.0200531.s003.pdf]

S3 Table. Comments received in response to technical and operational survey questions

**Area of survey**	**Examples of representative comments received** (Number of comments)
Treatment decisions to be guided by a new CA-LRTI POCT	(5)<br><br>'*initial treatment targeting - whether to prescribe an antibiotic or not'* should have been included in options'
Patient age group that a new CA-LRTI should benefit	(2)<br><br>'ideal test should be available for all age groups'<br><br>(4)<br><br>*'in neonates, we are very careful and start antibiotics easily when infection appears'*<br><br>*'if I was considering near patient testing in a child, I would probably be admitting them..'.*
Category of staff that would use the test most often	(8)<br><br>*'We have no nurses, practice nurse or nurse practitioner'*<br><br>*'Depends on the organisation of the healthcare system'*
Aetiological agents and antibiotic resistances for CA-LRTI POCT detection	(5)<br><br>'*I think it would depend on local prevalence'*<br><br>*'A test that could highlight an atypical organism may well have more clinical value..'*<br><br>*'Colonisation is important disturbing problem in microbiologic work-up'*
POCT and detection of antibiotic resistance	(3)<br><br>*'would depend on the country, and this would be directly related to my prescribing pattern'*<br><br>*'.. the ones we use! I haven't heard of half of the above!!'*
Test prediction and speed	(6)<br><br>*'Bearing in mind an average consultation in primary care is 10 mins.'*<br><br>*'Even shorter time periods, preferably within 3-5 minutes.'*<br><br>*'4 hours or less would be ideal'*<br><br>*'If not instant sub 4 hours is good'*
**Cost of instrumentation and test**	(6)<br><br>*'Maximum €500'* (for instrument)<br><br>*'even €10 would be too much'* (test)<br><br>'*€1 is the maximum acceptable test cost'*<br><br>*'depends on who is paying'*
