# Supplementary material for: Matching diagnostics development to clinical need: Target product profile development for a point of care test for community-acquired lower respiratory tract infection
Source: PLoS One. 2018 Aug 1;13(8):e0200531. doi: 10.1371/journal.pone.0200531 (PMC6070214; doi:10.1371/journal.pone.0200531)
Supplement: S4 Table — (PDF) [file pone.0200531.s004.pdf]

S4 Table. Clinicians perceived barriers to the uptake of a new POCT in primary care

**Area of potential barrier**	**Examples of comments**<br>(Number of comments)
**Time**<br><br>(time to obtaining the result, to take the test, and in relation to the clinical setting)	(10)<br><br>*'time constraints of primary care consultations particularly with multimorbidity'*<br><br>*'Time consuming'*<br><br>*'Workload'*
**Cost**<br><br>(cost of test and instrumentation, and in terms of cost effectiveness)	(19)<br><br>*'High cost of a POCT'*<br><br>*'Cost/benefit ratio = not convincing'*
**Clinical benefit**	(5)<br><br>*'..confidence that test would have a significant impact on management of a condition'*<br><br>*'scientific proof of benefit'*<br><br>*'limited added value'*
**Test complexity and ease of use**	(11)<br><br>*'Complexity of the rapid test'*<br><br>*'Difficult to use and/or interpret'*<br><br>*'Frequency of use. It has to be quite high, or we will never get used to it'*<br><br>*'Numbers for a complex test to be performed reliably'*<br><br>*'Difficulty of use'*
**Technical requirements of instrumentation and test**	(8)<br><br>*'Robustness'*<br><br>*' Not having next to patient'*<br><br>*'personnel training'*<br><br>*'quality control'*
**Performance and accuracy**	(7)<br><br>*'Reliability of the test results'*<br><br>*'Sensitivity / specificity'*
**Clinical samples and clinical pathway considerations**	(3)<br><br>*'Patient acceptability (if test is invasive)'*<br><br>*'Difficulty of getting sample(s) to practice'*<br><br>*'Sticking children'*
**Other**	(1)<br><br>*'Generally lack of widespread use of results in internal practice….. peer pressure to stick to traditional methods and abandon innovation'*
