# Supplementary material for: Matching diagnostics development to clinical need: Target product profile development for a point of care test for community-acquired lower respiratory tract infection
Source: PLoS One. 2018 Aug 1;13(8):e0200531. doi: 10.1371/journal.pone.0200531 (PMC6070214; doi:10.1371/journal.pone.0200531)
Supplement: S1 File — (PDF) [file pone.0200531.s005.pdf]

Point of Care Test for Community Acquired Lower Respiratory Tract Infection (CA-LRTI)

TARGET PRODUCT PROFILE (TPP)

(User Requirement Specification Document)

**This document has been authorised by:**			
**Name**	**Role**	**Signature**	**Date**
**Jorge Villacian**	**RAPP-ID coordinator**		
**Name**	**Role**	**Signature**	**Date**
**Herman Goossens**	**RAPP-ID Academic Coordinator**		
**Name**	**Role**	**Signature**	**Date**
**David Klenerman**	**Lead of Work Package 1**		
**Name**	**Role:**	**Signature**	**Date**
**Christopher Butler**	**Clinical specification requirements**		

Table of Contents

i	**Glossary of Abbreviations**	**2**
ii	**Definition of Point of Care Test**	**3**
1	**Source of document information**	**3**
2	**Aim and objectives of document**	**3**
3	**Clinical need**	**4**
4	**Need for new test**	**8**
5	**Clinical pathway and concept POCT diagrams**	**10**
6	**Current POCTs for LRTI**	**11**
7	**Notes on clinical samples**	**12**
8	**Diagnostic product specification (Intended use statement and test concepts)**	**14**
9	**Test Specifications table**	**15**
10	**Market Overview**	**20**
11	**Appendix 1<br>Competitor Tests<br>Definition of Test Outcomes**	**25<br><br>29**
12	**Appendix 2<br>Definitions of LRTI used in the survey**	**29**
13	**Appendix 3<br>Free text comments received in survey**	**30**
14	**References**	**34**

i. Glossary of Abbreviations

BAL	Bronchial alveolar lavage
CAP	Community acquired pneumonia
CFU	Colony forming units
CRP	C-reactive protein
DRSP	Drug-resistant *S. pneumoniae*
ELISA	Enzyme linked immuno sorbent assay
LRTI	Lower respiratory tract infection
NA	Nucleic acid
NP	Naso pharyngeal
PCR	Polymerase chain reaction
POCT	Point of care test
QCM	Quartz crystal microbalance

ii. Definition of Point of Care Test (POCT) for Primary Care

A POCT for primary care is defined as any test carried out in a primary care setting or the patient's home for which the result is available without reference to a laboratory and rapidly enough to affect immediate patient management. [1,2]

Primary care is the term for the health services (GP practices, health centres, accident and emergency services, GP out of houses centres, pharmacies) that play a central role in the local community. It refers to the work of health care professionals (general practitioner, family physician, pharmacist, nurse and other health care professionals) who act as a first point of contact for patients.

1. Source of document information

Key areas of relevance to end-users and information required by the POCT developers were identified through literature search, consultation with RAPP-ID partners, a web-survey targeted at practicing primary care clinicians (n=45. Participant countries: UK-21, Netherlands–3, Germany-1, Sweden-1, Spain-2, Australia-1, Belgium-9, Finland-1, Poland-3, Norway-1, and USA-2), and qualitative interviews with clinical trialists from industry and academia.

2. Aim and Objectives

The aim of this document is to:

Provide a reference document for the researchers, developers and manufacturers of the RAPP-ID Consortium

Objectives:

- Define the need for a novel POCT for Lower Respiratory Tract Infection in primary care.
- Define the user requirements for a POCT designed to aid in the identification of the usual organisms causing Community Acquired Pneumonia (CAP) in primary care, with a view to helping decide if immediate antibiotic prescription is necessary or not.
- To consider the need for a POCT for virus identification in primary are.
- Define the ideal and minimum acceptable product specifications for the CAP POCT.

3. Clinical Need:

A POCT test for Community Acquired Pneumonia (CAP) and Acute Cough in primary care: clinical considerations

CAP and Acute Cough can be both Lower Respiratory Tract Infections (LRTI). LRTIs are one of the commonest cause of acute illness in adults and one of the leading reasons for seeking medical care.

Figure 1. Spectrum of LRTI seen in primary care

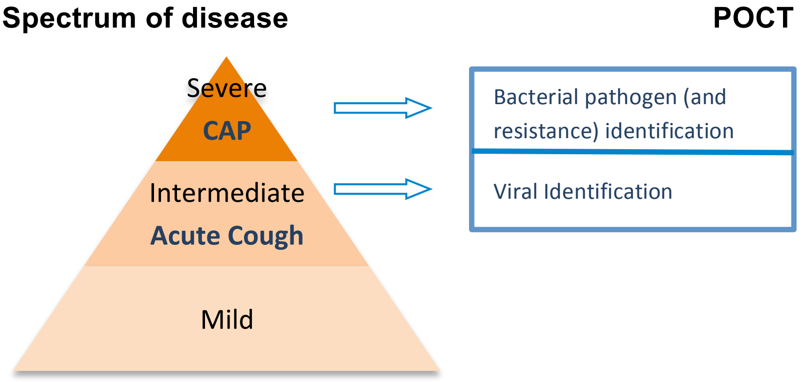

The need for a new POCT in primary care

The less severely ill

For the majority of patients presenting with mild to moderate illness in primary care, a test that gives prognostic information and rules out the need for antibiotic treatment would be most helpful. However, there is already a POCT for a biomarker, namely C-Reactive protein (CRP), and there are no known other biomarkers that have better prognostic properties in this setting. A *test to detect viral pathogens, which*, if known by a treating clinician to be present, might rule out the need to prescribe antibiotics and might rule in the need for antiviral treatment, would be the next most useful test for enhancing the management of these presentations in primary care. During pandemics, viral identification becomes more important as targeted antiviral therapy may be recommended. For example, during the recent H1N1 pandemic, more people received oselatmivir than were infected with the virus in primary and secondary care. As with overuse of antibiotics, over-use of antiviral agents is associated with developing resistance. An etiological diagnosis can guide decisions about other aspects of management apart from pharmacological treatment such as the decision about whether or not patients need to be isolated, and can help patients and clinicians make plans in the light of improved prognostic information.

The more severely ill

For the more severe end of the symptom severity spectrum, *a test to detect and identify bacterial pathogens and resistance factors* would be the most useful POCT. In patients assessed in hospital with possible CAP, a rapid POCT could influence patient outcome by aiding prompt imitation of appropriate antibiotics, with a favourable impact on mortality, antibiotic resistance, and cost (e.g. shortening the length-of-stay, decreased antibiotic consumption). The sooner appropriate antibiotic treatment is started in patients with CAP, the better the outcomes for patients. At the present time, two broad-spectrum antibiotic agents are usually started empirically for patients with CAP. Clinical outcomes and impact on resistance may be improved by a POCT that guides more specific therapy from the start of treatment. POCTs could also guide steeping down treatment to narrower spectrum (but equally effective) agents after treatment has already been started.

Focus on CAP

Incidence and cost: CAP is most often a bacterial infection and is has a high mortality (hospitalised patient mortality: 8% ward, 28% ICU). CAP is the leading cause of severe sepsis and death from infection in the United States.[3] CAP accounts for 600, 000 hospitalisations in the United States, with an annual cost in excess of €8 billion.
In the UK, the annual incidence of CAP is 5-11 per 1,000 adult population. The illness results in about 83,000 hospital admissions each year and is the fifth leading cause of death in the UK. The thirty-day mortality for all patients admitted to hospital with CAP is 18.3%, and these patients constitute 5.9% of critical care admissions.[4] Patients admitted to critical care have more severe disease, and accordingly critical care and hospital mortality rates are high (35% and 50% respectively).[5] The healthcare costs incurred as a result of CAP are estimated to be around €530 million annually in the UK. The average cost for management in the community was estimated at €120 per episode, and this can increase up to €6,123 when the patient is admitted to hospital. Substantial cost savings might be made by strategies for early. Well targeted effective therapy in CAP and thereby reduce hospital admission and improve patient outcomes.

CAP: A POCT to detect and identify bacterial pathogens and resistance factors: Which pathogens, how many, antibiotic resistances?

No two studies of the aetiology of CAP use the same procedures and produce consistent results. Observed differences in aetiology are due to healthcare setting, disease severity, population factors, and epidemics at the time of study (the prevalence of infections with some organisms is cyclical, and many vary at random from year to year), and depending on study methodology.

Etiological studies of CAP conducted in the UK.[6]

Organism	Setting where study was conducted		
	Community (1 study)	Hospital (Mean of 5 studies)	ICU (Mean of 4 studies)
Streptococcus pneumoniae	36.0	39.0	21.6
Haemophilus influenzae	10.2	5.2	3.8
Legionella spp	0.4	3.6	17.8
Staphylococcus aureus	0.8	1.9	8.7
**Moraxella catarrhalis**	?	1.9	?
**G-ve Enteric bacilli**	1.3	1.0	1.6
Mycoplasma pneumoniae	1.3	10.8	2.7
Chlamydophila pneumoniae	?	13.1	?
**Other**	3	5.8	7.1
All viruses	13.1	12.8	9.7
Influenza A+B	8.1	10.7	5.4
Mixed	11.0	14.2	6.0

The causes of CAP in other European countries are broadly similar to that in the UK. Studies of patients with severe CAP from other European countries suggest a lower frequency of Legionella and a higher frequency of Gram-negative enteric bacilli compared with the UK.[6] A test that detected a range of common bacterial pathogens would be of great value to clinicians, and there may be a case for the POCT to detect atypical pathogens (such as Legionella) as concern about these organisms leads some clinicians to prescribe more broad-spectrum antibiotics. However, following RAPP-ID workshop group discussions the decision was made to focus initially on the most common bacterial respiratory pathogen, namely *Streptococcus pneumoniae*.

A clinically useful POCT to enhance the management of possible CAP would need to quantify the *S. pneumoniae* bacteria order to differentiate colonisation (bacteria present but not causing diseases) from infection. Multiple studies of bacterial infections indicate that pathogens invariably reach 10^6 cfu/mL at infected sites (based on conventional microbiology culture). The clinical sample and method of collection must be taken into account. With quantitative cultures of expectorated sputum and endo-tracheal aspirates, the threshold is at least 10^6/mL, for brush catheter samples the threshold is 10^4/mL, and for Bronchial Alveolar Lavage (BAL) specimens the threshold is 10^3/mL.[7] (*Please see the 'notes on clinical samples' for quantification of *S. pneumoniae* from nasopharyngeal swabs). Including a test that measures patient inflammatory markers (produced when patient is infected) in sputum may an alternative option to organism quantification.

If antibiotics are prescribed, then prescribing antibiotics that are effective but with the narrowest spectrum to which the organism is sensitive is a generally agreed goal, as this is likely to limit the selection and spread of antibiotic resistant organisms. The emergence of drug-resistant *S. pneumoniae* (DRSP) is well documented. However, the clinical relevance of DRSP in CAP is uncertain. Studies indicate that current levels of resistance to β-lactam antibiotics generally do not result in treatment failure for patients with CAP when appropriate agents (i.e. amoxicillin, ceftriaxone and cefotaxime) and adequate doses are used, even in the presence of bacteraemia. However, macrolides are often prescribed for pneumonia and increasing macrolide resistance is now commonly seen in Southern Europe. There is evidence that resistance to macrolides may indeed result in failure of treatment with macrolide antibiotics.[8]

Focus on Acute Cough

Cough often presents to clinicians working in primary and secondary care simply as a troublesome symptom . Acute cough is usually due to an infectious organism (bacteria or virus) though there are also non- infectious causes. The vast majority of cases of acute cough presenting to primary care are due to acute viral respiratory tract infection. While many episodes of acute cough are self-limiting and generate little diagnostic uncertainty and can easily be managed without recourse to diagnostic tests, antibiotics continue to be over prescribed for this condition. In the face of increasing diagnostic uncertainty, clinicians tend to prescribe empirical antibiotics. A major worry for them is to 'miss' a case of pneumonia. Estimates vary, but around 5% of patients with acute cough have pneumonia. A test that rapidly differentiates these patients with pneumonia from those that are unlikely to benefit from antibiotic treatment would greatly benefit clinicians.

Incidence and cost

Acute cough is one of the most common symptoms for which patients seek medical attention, the most common new presentation in primary care, and the most frequent reason for visits to hospital-based outpatient clinics. Approximately 100/1000 of all people in developed countries will present to primary care with an acute cough, and 2/1000 will require hospital treatment. In the USA, acute cough accounted for 26 million office visits in 2004.[9] It is estimated that he cost of acute cough to the UK economy is estimated to be at least €1,175 million/year. This comprises €1.050 million to loss of productivity and €125 million cost to the healthcare system and the purchase of non-prescription medicines.[10] In the UK, annual prescribing costs for acute cough alone exceed €18 million.[11]

4. The need for a new test

It is difficult for clinicians on clinical grounds to differentiate with adequate precision between 'less severe' LRTIs and CAP, as early clinical features can be similar. Currently, no clinical features or current diagnostic test, alone or in combination, adequately determine diagnosis, aetiology, prognosis, or response to treatment.

Between 20%-95% of patients presenting with suspected LRTI in the community receive antibiotics and it is estimated that 80% of these antibiotics may be unnecessary. Antibiotic use exposes patients to risk of side effects and is associated with antibiotic resistance. A number of interventions, including point of care tests (POCT), can reduce antibiotic prescribing to patients by clinicians. However, this reduced prescribing may also be related to an increasing incidence of CAP. Without a POCT that performs well, clinicians cannot rapidly enough distinguish viral from bacterial causes, and which patients require antibiotics and which patients can safely be treated without antibiotics. Often, clinicians are unwilling to withhold antibiotic treatment without evidence that a patient may not be subjected to harm. A POCT that could tell a clinician if the acute cough is due to a bacterial or viral cause (and/or not requiring antibiotic) could reduce unnecessary antibiotic prescriptions and reduce antibiotic resistance.

Making a difference to clinical outcomes and feasibility n everyday care

Before a new POCT can be recommended for use in routine care, there should be data demonstrating improved patient outcomes from clinical decision making that incorporates POCT results. Without this, POCTs will simply add to process complexity and generate unnecessary cost. There are already a number of diagnostic POCTs available for CAP and LRTI, but many of these tests do not meet a real clinical need and are often not feasible for practical use in everyday primary care.

Apart from being able to demonstrate improved patient outcomes or cost effectiveness form POCT guided management, POCTS need to be feasible for use in primary care for optimal uptake into routine care. For example, clinicians and patients will not use tests that are too expensive, time consuming, hazardous, invasive, bulky, and maintenance intensive. If we hope to successfully integrate a POCT into a clinical pathway we need also to carefully consider both clinician and patient's views and requirements of POCT.

Qualitative research among clinicians in primary care indicates that they favour a test that is cheap, accurate (in the milder cases, ruling out the need for antibiotics is most important), requires minimal training, maintenance, storage, calibration, and that the time to result should be very short. A test should be able to produce a result in the time people might reasonably be expected to wait in a busy general practice surgery during a time they are ill. Typically, a clinician may take a test sample, asks the patient to wait in the waiting room, then see another patient (possibly two patients), and then see the original patient again to give the test result and decide on management. Such a time frame would typically between 10 and 20 minutes but certainly within 30 minutes.

Acute cough: A POCT to detect and identify common respiratory pathogens:

Detection of common respiratory bacterial and viral pathogens from a naso-pharyngeal (NP) swab or a breath sample would be of value in a primary care setting for patients with less severe illness where the decision about prescribing antibiotics was uncertain. Detection viral and bacterial pathogens is likely to help in decisions about antibiotic prescribing, may help in reassuring patients, and may be of help in making specific treatment decisions (for example antivirals for treating influenza).

The rapid influenza component of the test would be valuable in everyday primary and secondary care (particularly during the influenza season), in pandemic situations, and in settings outside of the healthcare system, such as ports of entry.

5. Outline of clinical pathway showing possible use of a POCT in primary care

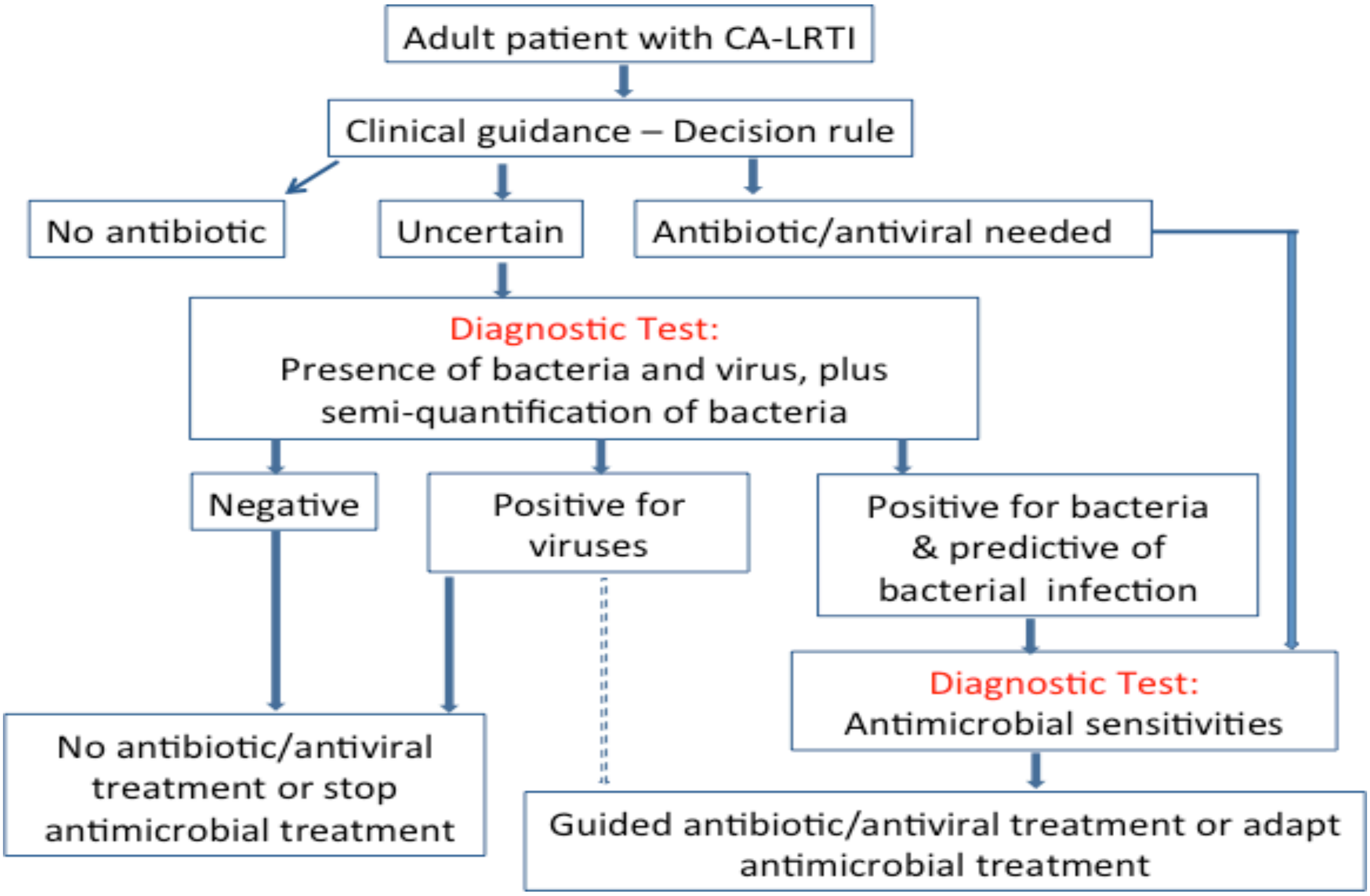

Diagram of POCT Concept

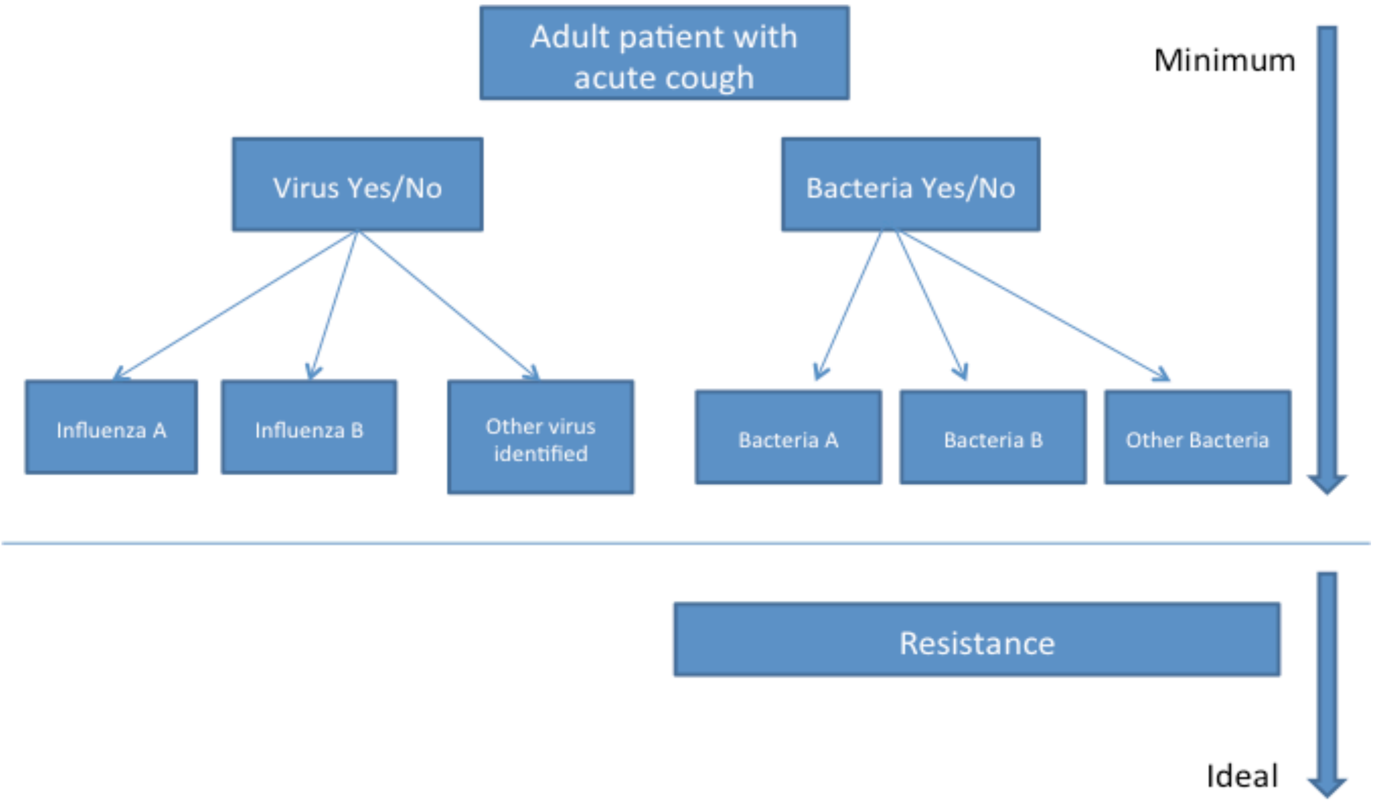

6. Current POCTs for CA-LRTI

There are many POCTs already available to aid the diagnosis of Lower Respiratory Tract Infections (LRTI) in primary care. These include the ArcDia mariPOC® multi-analyte test targeting respiratory viruses and *Streptococcus pneumoniae*, and the BinaxNOW *S. pneumoniae* urinary antigen test and numerous influenza tests (see Appendix 1). However, despite the availability of many POCTs, they are seldom used in routine clinical care in most countries. There are clinical niches where better POCTs are urgently required. It is known that the BinaxNow test has insufficient sensitivity. However, the mariPOC® system appears to meet many of the requirements of a POCT for primary care use. However, we are unaware of its currently being widely used in this setting. This could be due to factors such as the cost of the analyser or tests, size of the analyser, unacceptability of patient sample and should be explored.

A number of influenza rapid diagnostic tests exist for the screening for influenza virus infection and these can provide a result within 15 minutes. Some of these tests can identify influenza A and B viruses and distinguish between them, whereas others can identify influenza A and B viruses but cannot distinguish between them. Most of these tests can be used with a variety of specimen types, but the accuracy of the tests can vary based on the type of specimen collected (for example throat swab versus nasal swab). The rapid tests vary in terms of sensitivity and specificity when compared with viral culture or RT-PCR. Product insert information and research publications indicate that sensitivities are approximately 50-70% and specificities are approximately 90-95%. Specimens to be used with rapid tests generally should be collected as close as is possible to the start of symptoms and usually no more than 4-5 days later in adults. In very young children, influenza viruses can be shed for longer periods; therefore, in some instances, testing for a few days after this period may still be useful. The accuracy of the influenza tests depends on the prevalence of influenza, and the positive and negative predictive values vary considerably depending upon the prevalence of influenza in the community. False-positive (and true-negative) influenza test results are more likely to occur when disease prevalence is low, which is generally at the beginning and end of the influenza season. False-negative (and true-positive) influenza test results are more likely to occur when disease prevalence is high, which is typically at the height of the influenza season.

Rapid methods for the detection of other potentially pathogenic respiratory viruses

In general, rapid methods for the detection of respiratory viruses are based either on immunological, or on molecular techniques. The majority of immunological methods for respiratory viruses are based on antigen detection and use immunofluorescence, enzyme immunoassay (EIA), or immunochromatography technologies. A number of commercial products are available, including tests for the detection of up to seven different respiratory viruses in culture. Commercial immunoassay-based products have also been developed as tests that can be applied at the point-of-care. A wide range of lateral flow

immunochromatography test strips is available for the detection of influenza A & B viruses and RSV in clinical samples. These tests are simple to use and can be performed outside of the laboratory to provide a rapid diagnosis. Their main disadvantages are that they are usually only qualitative tests, and have limited sensitivity. Immunoassay methods based on antibody, rather than antigen, detection are also available for the detection of respiratory virus infection.

Most molecular techniques rely on detecting specific viral RNA sequences. The molecular methods can improve specificity and sensitivity significantly. Real-time PCR also has the advantage of allowing the virus to be quantified. Multiplex PCR methods have been developed that allow the detection of more than one nucleic acid sequence, and therefore more than one virus type, in the same assay. Other nucleic acid amplification methods have also been developed, notably nucleic acid sequence base amplification (NASBA). This technique is also known as isothermal amplification, as the temperature remains constant throughout. A commercial NASBA test has been developed for metapneumovirus detection. Nucleic acid amplification techniques are extremely sensitive which also makes them vulnerable to contamination. Most molecular methods also require some costly equipment and are not suitable for use outside the laboratory. Non-quantitative PCR results may also be difficult to interpret, since low numbers of virus that do not signify an infection may be detected.

7. Notes on Clinical Samples

Intuitively, it seems that organism detection may be optimal from a breath test rather than nasopharyngeal (NP) swabs (NP). A breath test would also be optimal for ease of use and patient acceptability, as it is one of the least invasive way of sampling. It does not require blood, sputum, urine, or pharyngeal or nasopharyngeal swab (i.e. non invasive). Clinician and patient acceptability is a major concern for successful uptake of any POCT especially in those with milder illness in primary care, and nasopharyngeal washings were found to be more acceptable than nasopharyngeal swabs in 91% of participants in one study.[12] Additionally, over 30% of the clinicians consulted in the RAPP-ID survey stated that they would not use a test that required a nasopharyngeal swab to be taken, though this may be due to unfamiliarity with the technique. However, nasopharyngeal swab samples were found to be well tolerated in the FP6 funded Genomics to combat Resistance against Antibiotics in Community-acquired LRTI in Europe (GRACE) Network of Excellence Randomised Controlled Clinical Trial of amoxicillin versus placebo for acute cough (Goossens, personal communication) and so swab type and clinician/nurse training may be an important issue here.

In addition to causing CA-LRTIs, *S. pneumoniae* can also colonise healthy people and has been found in the nasopharynx of 5-20% of healthy adults, and in up to 50% of healthy

children. Quantitation of pneumococci in nasopharyngeal samples is promising as method for distinguishing asymptomatic colonisation from active infection and may be predictive of pneumonia. Patients with CA-LRTI showed 28-fold higher average loads of *S. pneumoniae* than colonised individuals.[13] Results (using nasopharyngeal swab samples) obtained with a semi-quantitative culture method showed good correlation with real-time PCR, although this study was small. However, it is difficult to clearly define an quantification threshold that differentiates invasive pneumococcal disease from colonisation and so the practical use of this measurement should be further evaluated.[14] Despite this, it should be considered that the prescription of antibiotics to a person 'colonised' rather than infected with *S. pneumoniae* is perhaps of less importance than the number of patients treated with antibiotics for a viral infection. The threshold for quantitative cultures from nasopharyngeal aspirate for diagnosing probable pneumococcal pneumonia has been quoted as $\geq 10^6$ cfu/ml [15] and $\geq 10^5$ cfu/ml. [16]

Exhaled breath condensate (EBC) has been used for the detection of bacteria including *S. pneumoniae* from patients with acute exacerbation of chronic obstructive pulmonary disease (COPD).[17] However, PCR analysis found that EBC and sputum results did not correlate well in this study though this may be related to novel technology that is still in development. Specific Volatile Organic Compounds (VOCs) can be detected in the exhaled breath of patients with exacerbations of COPD,[18] and volatile metabolites specific for *S. pneumoniae* have been isolated when the organism is grown in culture.[19,20] However, no references were found for *in vivo* detection of VOCs from *S. pneumonia*e.

Therefore, data are needed for both types of sample to differentiate colonisation from lower airway infection.

8. Diagnostic product specification (Intended use statement)

DIAGNOSTIC PRODUCT SPECIFICATION
**Intended Use(s) of the test:**

- A POCT to enhance the initial clinical management of suspected CAP in adult patients presenting in primary care. The POCT should achieve this by indicating with sufficient precision the presence or absence of the organisms that account for almost all cases of CAP, so as to inform decisions about whether an immediate antibiotic prescription is necessary or not.
- The primary target population is in primary care.
- The POCT should be easy to use by a range of health care professionals in a variety of settings (e.g. doctors, nurses and any community health workers in any primary care setting doctors surgery, GP home visit, and nursing homes).
- The POCT should be feasible and cost effective in the primary care setting.
- The test is designed to be used with either nasopharyngeal (NP) swab or exhaled breath samples without need for prior culture.

Test Concept:

- The POCT should be able to detect *S. pneumoniae*, *H. influenzae, S. aureus, M. catharalis, Chlamydophila pneumoniae, Mycoplasma pneumoniae, Legionella spp and common viral respiratory pathogens including parainfluenza, rhinovirus, respiratory syncytial virus (RSV) and influenza A and B, coronaviruses, human metapneumovirus, respiratory syncytial virus (RSV) and parainfluenza.*
- *The POCT should be able to detect* penicillin and macrolide resistance in common potentially pathogenic respiratory bacteria.
- The POCT should provide a technologically flexible platform that allows the future incorporation of biomarker detection, should biomarkers be identified that will add to diagnostic performance.

Proof of Concept:

- A POCT to identify *S. pneumoniae, H. influenzae, S. aureus, M. catharalis, Chlamydophila pneumoniae, Mycoplasma pneumonia, Legionella spp and common viral respiratory pathogens including rhinovirus, influenza (A & B), coronaviruses, human metapneumoviruses, respiratory syncytial virus (RSV) and parainfluenza.* The results of the POCT should be used to accurately identify the presence of common pathogenic respiratory bacteria and viruses in adult patients presenting with symptoms of CA-LRTI, to rapidly guide clinicians in their decisions whether or not to prescribe antibiotic or antiviral treatment.

9. Test Specifications Table

KEY FEATURES		
**TECHNICAL SPECIFICATIONS**	**Ideal (Target)**	**Minimum (Acceptable)**

**Intended use**	To aid the antibiotic and antiviral prescribing and treatment decisions for adults presenting with symptoms of CA-LRTI in any primary care setting including nursing homes, care homes, out-of-hours clinics, accident and emergency departments, GP practices etc.	To aid the antibiotic and antiviral prescribing and treatment decisions for adults presenting with symptoms of CA-LRTI in the primary care setting (clinician surgery).
**Medical decision to be influenced**	Whether or not to prescribe and antibiotic or antiviral agent for patients with CA-LRTI, and if so, which agent to select for maximum patient benefit and minimal impact on selection of antimicrobial resistance	To guide clinical decision making, specifically about whether or not to prescribe antibiotic and antiviral agents, for the common causes of CA-LRTI.
**Place of use**	Any site where a patient may consult within the primary care setting (including nursing homes, care homes, out-of-hours clinics, accident and emergency departments, GP practices) and elsewhere.	Any site where a patient may consult within the primary care setting.
**Patient criteria**	All adult patients including antibiotic pre-treated and those with no prior antibiotic treatment for that episode of CA-LRTI.	All adults with no previous antimicrobial treatment for that episode of illness

**TECHNICAL SPECIFICATIONS**	**Ideal (Target)**	**Minimum (Acceptable)**
**Target molecule** (Analytes to be detected)	Pathogens: the most frequent respiratory pathogens including *S. pneumoniae*, H. *influenza, S. aureus, M. catharalis, Chlamydophila pneumoniae, Mycoplasma pneumonia, Legionella spp* and a comprehensive (8-10) number of common viral respiratory pathogens.<br><br>Antibiotic resistance genes: Macrolide and penicillin resistance genes in common potentially pathogenic respiratory bacteria.<br><br>(Biomarkers as they become available and are shown to have added diagnostic value).	Pathogens: The most frequent respiratory pathogens including *S. pneumoniae*, H. *influenzae, S. aureus, M. catharalis, Chlamydophila pneumoniae, Mycoplasma pneumoniae, Legionella spp* and (6) common viral respiratory pathogens including rhinovirus, influenza A and B, coronaviruses, human metapneumovirus, respiratory syncytial virus (RSV), and parainfluenza.

**Sensitivity**<br>The ability of the test to correctly identify those patients with the disease.<br>Independent of the population of interest subjected to the test.[23]<br>Sensitivity= True positives / True positives + False negatives	- 100%	>85%<br><br>(In primary care, the sensitivity of GP clinical diagnosis alone in predicting radiographic confirmed pneumonia is 60%.[21])
**Specificity**<br>The ability of the test to correctly identify those patients without the disease.<br>Independent of the population of interested subjected to the test.[23]<br>Specificity= True negatives / True negatives + False positives	- 100%	>85%<br><br>(In primary care, the specificity of GP clinical diagnosis alone in predicting radiographic pneumonia is 68%.[21])
**Positive predictive value (PPV)**<br>Important for clinicians: Answers question "How likely is it that this patient has the disease given that the test result is positive?"<br>PPV is dependent on the population being tested and the prevalence of disease.[23]<br>PPV = True positives / True positives + False positives	100%	>85%?<br><br>(In primary care, the PPV of General Practitioner clinical diagnosis alone for radiographic pneumonia is 23%, rising to 32% if CRP ≥20mg/l is added as a diagnostic criterion, but the latter is at the expense of a lower sensitivity of 49%.[21]).<br><br>(GP clinical diagnosis of influenza virus infection: PPV is 76% (for a combination of clinical signs.[22])
**Negative Predictive value (NPV)**<br>Important for clinicians: Answers question "How likely is it that this patient does not have the disease given that the test result is negative?"<br>NPV is dependent on the population being tested and the prevalence of disease.[23]<br>NPV = True negatives / True negatives + False negatives	100%	>91%<br>(In primary care, the NPV of GP clinical diagnosis for radiographic pneumonia is 91%, and remains at this level if CRP ≥20mg/l is added.[21]).<br><br>GP clinical diagnosis of a microbiologically proven influenza virus infection: NPV is 75% (for a combination of clinical signs.[22]).
**Type of analysis**	- Qualitative and quantitative analysis (or semi-quantitative analysis of pathogens present as both colonisers and infective agents) of the above mentioned bacterial pathogens.<br><br>- Qualitative analysis of common respiratory viruses including influenza A and B.	- Qualitative detection of *the listed bacterial pathogens (For S. pneumoniae the boundary for detection should be set at a level which is indicative of colonisation rather than infection.*<br><br>- Qualitative detection of common respiratory viruses

	- Qualitative analysis of S. *pneumoniae* macrolide and penicillin resistance genes.	including influenza A and B.
**Reading system and Result (post) readout**	- Easy to read, unambiguous "yes", "no", "inconclusive" (for quantifiable result) or "'invalid" answer for each individual pathogen.<br><br>- Readable for at least 1 hour.<br><br>- Result downloadable to patient electronic records ("connectivity").	- Easy to read, unambiguous "yes", "no", "inconclusive" (for quantifiable result) or "'invalid" answer for each individual pathogen.<br>(Note: The above may need to be modified in light of technical specifications)<br><br>- Readable for at least 1 hour.
**Sample type**	Exhaled breath	Nasopharyngeal swab

REPRODUCIBILITY	Ideal (Target)	Minimum (Acceptable)
**Reproducibility**<br>Conditions where test results are obtained with the same method on identical test material in different laboratories with different operators.	100%<br><br>(Fully compliant with FDA and EU guidance including Waived Status.	>95%<br><br>(Fully compliant with EU regulatory requirements).<br>(A non-Waived status product, even one with FDA compliance may still be very useful in the EU).
**Reproducibility near clinical threshold**<br>Gives some idea of the chance of correctly classifying borderline specimens by testing a panel of well characterised and representative samples close to the clinical *threshold	>95%<br><br>*(Need to consider possible confounding factors and test to determine if problem or not).	>90%

Operational characteristics	Ideal (Target)	Minimum (Acceptable)
**Volume of sample required**	- 1 sample per test.<br>- Usual volume range obtained from 1 nasopharyngeal (NP) swab using standard method.<br>For breath test: usual volume in a single exhaled breath	- 1 sample per test.<br>- Usual volume range obtained from 1 NP swab using standard method.<br>For breath test: any usual volume in exhaled breaths.
**Sample preparation**<br>Requirement to process sample prior to testing	- No sample preparation steps prior to testing.	- 1 preparation step maximum (22% of clinicians in our survey indicated they would not use a test that

		requires more than one simple preparation step). Manual elution of swab into buffer solution may be acceptable.
**Requirement for precise volume** (sample / reagent)	Ability to use approximate volumes of sample and reagent (i.e. no need for precise pipetting).	Ability to use approximate volumes of sample and reagent (i.e. no need for precise pipetting).
**Time to result**	< 10 minutes (i.e. could be concluded in a single, typical general practice consultation time).	< 30 minutes.<br>(Could be longer if incorporated into a 'delayed antibiotic prescribing' pathway. But this sort of test may require different specifications then those in this document).
**Test stability**<br>Temperature etc at which test should remain stable for a defined length of time	Stable at well beyond ambient room temperatures and any temperatures that may be encountered in the shipping process.	Stable at ambient room temperatures normally encountered and any temperatures that may be normally encountered in the shipping process.
**Storage conditions**<br>Prior to utilisation	Storage at well beyond ambient room temperature and humidity.	Storage at ambient room temperature and humidity.
**Shelf-life of reagents**	Shelf life of >12 months, including reagents.<br>(These should meet the regulatory requirements of EU and FDA)	Shelf life of >6 months, including reagents.<br>(These should meet the regulatory requirements of EU and FDA)
**Shelf life of instrument**	This instrument should ideally be usable without maintenance forever (>5 years).<br>(These should meet the regulatory requirements of EU and FDA)	At least 3 years.<br><br>(These should meet the regulatory requirements of EU and FDA)
**Test requirements**	- Must be totally self-contained (including sample collection vessel)<br>- No reconstitution requirement.<br>- Requires no calibration before each test	- Must be totally self-contained (including sample collection vessel)<br>- Simple reconstitution only.<br>- Requires no calibration before each test
**Controls**	- Positive and negative control included in the kit and as a part of each test.<br>- No external quality control needed.	- Positive and negative control included in the kit and as a part of each test.<br>- Quality control simple and not frequent.
**Waste disposal**	- Simple sharps disposal and any other disposal must be suitable for primary care setting.<br>- No glass component.<br>- Environmentally acceptable	- Simple sharps disposal and any other disposal must be suitable for primary care setting.<br>- No glass component.<br>- Environmentally acceptable

	disposal and fit in with existing arrangements for clinical waste disposal in primary care.<br>- Should comply with Waste Electrical and Electronic Equipment (WEEE) Directive and other environmental directives and regulations.	disposal.<br>- Should comply with Waste Electrical and Electronic Equipment (WEEE) Directive WEEE and other environmental directives and regulations.
**Test throughput/ batching**	1 test at a time would need to be feasible.<br>(Tests would probably not be batched, however a modular expansion should be possible, like Enigma, Smiths and Cepheid).	1 test at a time would need to be feasible.
**End user profile**	Can be used by any health care worker (doctors and any nurses).	Doctors
**Training**	No training. (Fully intuitive and self-training provided with instrument use and demanded by instrument to be completed. A biometric identification system can assist with this.)	Maximum 1 day training time.
**Biosafety requirement**	Safe: No bio safety issues with running test or disposal following use.	Safe: No bio safety issues or disposal following use.
**Instrumentation requirement**	- Hand held<br>- Easy to transport (e.g. take to nursing home consultation)<br>- Shock resistant as tested according to defined standard;<br>- Easy to clean/decontaminate	- Smaller footprint than a typical laptop / notebook computer;<br>- Volume <5L, weight <10kg;<br>- Shock resistance aspects taken into consideration during design and construction (best effort) but no formal test;<br>- Easy to clean/decontaminate
**Need for additional equipment**	No requirement for any additional equipment.	No requirement for additional automated equipment (e.g. vortexers, centrifuges).
**Maintenance of instrumentation**	No maintenance.	Annual maintenance at most.
**Power requirement**	Can work on battery and mains.	Can work on mains.<br>Max power 300W<br>Max start-up current from 12V, 15A
**Cost of test to <u>end user</u>**	Considerably less than €10 per test.	No more than €10 per test.<br>More allowed for clinical trials on case-by-case review but still less than €200 per test.

**Cost of platform to end user**	Free or leased as part of a per use contract.<br><br>(For reference, Cepheid GX-I lists at €10,000 but actually sells for around €8,500)	Less than €2,000 for commercial sales but provided free for clinical trials for the duration of the trial.

10. Market Overview

Market Overview	Ideal (Target)	Minimum (Acceptable)
**Cost of manufacturing single use device**	Less than €2 per test when established volume of much greater than 10,000 units per annum is achieved.	€4-8 per test with established volume of 10,000 units per annum.
**Competitive landscape**	The need ideally, is to provide: a much faster – less than 10 minutes turn around time (TAT), better PPV and NPV than current techniques and competitors, and at a much lower price.<br><br>The large established players currently focussed on the laboratory market must be considered as possible competitors and likely to be able to leverage on their large volumes in the central laboratory / hospital markets to lower overall test costs. Relatively simple lateral flow tests also create an expectation of fast, low cost solutions to serve this market. The performance of such tests is unlikely to be challenged in the primary care setting as much as within the hospital or laboratory setting.	The performance of Cepheid's GeneXpert is the current benchmark for many POC molecular tests and an important target to beat.<br><br>Smiths Detection Diagnostics and Engima's ML are the other two emerging players to monitor for performance both clinically and commercially since they should be able to serve the market competitively. All three of these products are modular and scalable and so can also address the hospital based POCT opportunity.
**Regulatory and legal pathway**	EU CE Marking and FDA Premarket Notification 510(k) potential demonstrated and FDA Premarket Approval if required.<br>Analytical results validated by prospective clinical investigations / performance evaluations.<br>Design process and potential for manufacturing scale up	EU CE marking potential demonstrated, working to appropriate GHTF risk classification requirements, i.e. High Individual Risk and/or Moderate Public Health Risk.<br>Initial performance evaluation using analytical results. Design process fully documented.

	fully documented.	
**Region(s) of commercialisation**	EU, North America, Japan, China, India and Argentina.	EU and markets that have a mutual recognition agreement (MRA) or accept CE marking for market access, e.g. Australia and New Zealand.
**Market Segmentation**	Any primary care healthcare setting for respiratory pathogen identification and drug resistance determination. Primary use targeted at both routine GP use and to assist in making CA-LRTI drug trials more efficient and effective. Secondary use targeted at antimicrobial stewardship.	High resource community setting, where no laboratory skills are available, to test for the presence of specific respiratory pathogens. Use targeted at both routine GP use and to assist in making CA-LRTI drug trials more efficient and effective.

It is noted that according to the accounting consultancy PwC and Roche the global molecular diagnostics market was worth US$3 billion during 2009 but will double in size to around US$6 billion by 2014 and is the fastest growing segment of the IVD sector. This figure takes no account of the added value from assisting companion pharmaceuticals or other therapeutics remain or reach the market and provide huge social and economic benefits. The overall commercial payback in such cases can be ten times the revenues of the diagnostic.

Mergers and acquisitions are currently considered to be at exceptional levels and reflect the need of pharmaceuticals to invest in diagnostics to ensure sustainable business models emerge for the future. It is expected, given current trends, that by 2020 the need for co-development of drug and diagnostics will drive all the major pharmaceutical companies to acquire or develop an in-house diagnostic capability. The trends in business models can neatly be summarised as a move from blockbusters to niche-busters.

Early detection of disease and personalised medicine are the key areas of current activity and look set to be a significant part of new emerging healthcare delivery models. The desire of governments is move as much care and management of patients to the community as possible and keep costs as low as possible by ensuring early interventions are truly cost effective whilst delivering improvements in morbidity, mortality and patient quality of care.

Market Overview Useful References

ECRI Institute Health Technology Assessment Intelligence Report for molecular based POCT of LRTI provided to Medical Device Consultancy (MDC) as a custom report during January 2010. The copyright remains with ECRI Institute. The report includes information concerning GeneXpert, bioMerieux, HandyLab, Osmetech and others. The report is evidence based and includes research findings concerning:

- Rapid test to screen for methicillin-resistant Staphylococcus aureus (MRSA) that provides an overview, technology description, care setting, background on the disease/condition, prevalence/incidence rate, manufacturers/suppliers, regulatory status, reported patient indications/contraindications, clinical practice guidelines/other evidence reports/standards /guidelines and most importantly:

- Considerations for Hospitals:
- Impact on Hospital Operations
- Safety
- Credentialing/Training
- Competing and Complementary Technologies including Effect on Other Technologies
- Diffusion Status including Phase of Diffusion and Ongoing Clinical Trials
- Cost and Reimbursement including Cost Effectiveness & Considerations
- Evidence/Outcomes including Reported Outcomes/Adverse Events
- Summary/References
- Rapid diagnostic test to detect influenza provides similar information to that indicated
- above.
- Rapid swine flu tests under rapid development.
- Test for avian influenza provides a useful overview (as above) includes impacts, test prices and ECRI perspectives and predictions.
- Clinical Utility of Methicillin-resistant Staphylococcus Aureus Screening Tests provides a summary on clinical utility and has a useful review of clinical studies and extensive references.

- Internet-based infectious disease surveillance systems for predicting and/or tracking disease outbreaks and spread provides a very useful review of key issues that are highly relevant to RAPP-ID.
- Invader UGT1A1 Molecular Assay for Personalized Medicine Management document
- provides some initial insights into areas that might suggest future use areas of application for RAPP-ID technology such as pharmacogenetics and metabolomics. Molecular-based Diagnostic Testing Using PathFinderTG is not currently relevant to RAPP-ID but does provide further insights into personalized medicine that should eventually be of real interest.
- The PCR (Polymerase Chain Reaction) Test for Diagnosis of Lyme Disease is included for completeness and does give a benchmark on sensitivity specificity, PPV, NPV of various players – this is the sort of analysis that any major laboratory is likely to undertake for themselves.
- Diagnostics 2011: M&A surges, companion diagnostics accelerate, and early detection offers new prospects. PWC, December 2011.
- Hospital-Acquired Infection Diagnosic Market and Test Developments, Treatment and Trends. Kalorama Information, July 2011.
- World Health Organization (WHO) web site on infectious diseases, including drug-resistant tuberculosis and other infectious diseases.

11. Appendix 1.

11.1 Competitor tests (based on current manufacturers marketing material):

Information is provided here about the best currently available tests in order to guide the development of tests that will be more useful.

1. ArcDia mariPOC® Test: Multianalyte, point-of-care, immunoassay test system

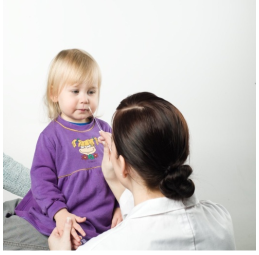
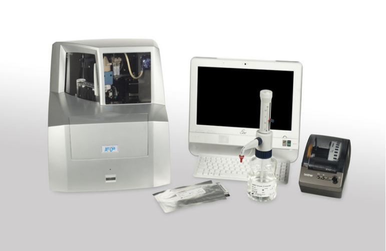

for testing of respiratory tract infections.

Performance as specified by test manufacturer:

**Sensitivity**	Superior to rapid tests and ELISA, similar to DELFIA
**Specificity**	Superior to rapid tests, similar to ELISA and DELFIA
**Turnaround time**	20 minutes for high positive and positive samples for low positives and negative samples
**Through-put time**	50-100 multianalyte tests per day
**Hands-on-time**	1 minute
**Cross-reactivity**	No cross-reactions found against the most common respiratory pathogens

(DELFIA: Dissociation Enhanced Lanthanide Fluoroimmunoassay).

Performance figures provided by manufacturer:

**Analyte**	**Sensitivity**	**Specificity**	**N**	**Reference method**
**Influenza A virus**	100%	100%	102	DELFIA
**Influenza B virus**	Similar to 10 ng antigen/ml	No cross-reactions	NA	DELFIA
**Adenovirus**	92%	100%	95	DELFIA
**Respiratory syncytial virus**	100%	100%	94	DELFIA
**Human metapneumovirus**	Similar based on sample dilution studies	100%	43	Biotrin ELISA
**Parainfluenza virus 1**		100%	55	DELFIA
**Parainfluenza virus 2**		98%	55	DELFIA
**Parainfluenza virus 3**		100%	55	DELFIA
Streptococcus pneumoniae	Similar 200CFU/ml 10ng antigen/L	No cross-reactions	NA	Binax Now! Lateral flow
**Group A streptococci**	>100%	100%	427	Culture, lateral flow

Choice of three test panels:

MariPOC combi: Combines all the above pathogens in a single test (Sample is a nasopharyngeal swab)

MariPOC respi: 9 parameter test including all above organisms except GroupA strep from a nasopharyngeal swab.

MariPOC pharyn: 2 parameter pharyngitis test (Adenovirus and Group A Strep) from a throat swab.

2. BinaxNOW *Streptococcus pneumoniae* urinary antigen test

The BinaxNOW *Streptococcus pneumoniae* antigen test (Binax, Scarborough, Me, USA) is a rapid ICT assay validated for urine and cerebral spinal fluid samples. A swab is dipped in the urine and then placed on a nitrocellulose membrane that contains rabbit antibodies against *S. pneumoniae*. Six drops of a supplied reagent solution are added, and after 15 minutes the card is read. There is one line for the internal control, and the appearance of a second line signifies a positive result. The test detects the C-polysaccharide from the cell wall of all pneumococcal serotypes.

Sensitivity is 70%–92% for patients with bacteraemic pneumococcal pneumonia and is at the higher end of the range for those with more severe infection. Timing of the test in relation to disease onset is not critical, as the test can remain positive for a month or more after pneumococcal infection. Specificity is higher than 90%.The test is useful for diagnosing pneumococcal infections rapidly — particularly in people who have already received antibiotics as, unlike cultures of blood and sputum, antibiotics do not appear to affect the test's accuracy. The test appears to be less useful in children because nasopharyngeal colonisation with *S. pneumoniae* can lead to false positive results. A more recently identified use of the pneumococcal antigen assay is in the diagnosis of *S. pneumoniae* meningitis, when it can be used on cerebrospinal fluid rather than urine. In this setting, it can detect pneumococcal meningitis with sensitivity and specificity of over 95%.

Manufacturers information

A negative BinaxNOW® Test does not exclude infection with *S. pneumoniae*. Therefore, the results of this test as well as culture results, serology or other antigen detection methods should be used in conjunction with clinical findings to make an accurate diagnosis. The BinaxNOW® *Streptococcus pneumoniae* Test has not been evaluated on patients taking antibiotics for greater than 24 hours or on patients who have recently completed an antibiotic regimen. *Streptococcus pneumoniae* vaccine may cause false positive results in urine in the BinaxNOW® *Streptococcus pneumoniae* Test in the 48 hours following vaccination. The effect of vaccination has not been determined on persons with pneumococcal meningitis. Hence, it is recommended that the BinaxNOW® *Streptococcus pneumoniae* Test not be administered within 5 days of receiving the *S. pneumoniae* vaccine. The accuracy of the BinaxNOW® Test in urine has not been proven in young children. Performance on CSF in young children, on the other hand, is established.

PERFORMANCE DATA - URINE

ANALYTICAL SENSITIVITY

Serotype Evaluation

Forty-four (44) isolates, representing the 23 *S. pneumoniae* serotypes responsible for at least 90% of serious pneumococcal infection in the United States and worldwide, were grown in culture and found to be positive in the BinaxNOW®
Test at concentrations of 10^5 cells/ml.

Limit of Detection

The BinaxNOW® Test limit of detection (LOD), defined as the dilution of positive urine that produces positive BinaxNOW® Test results approximately 95% of the time, was identified by preparing multiple dilutions of a known positive patient urine and running these dilutions in the BinaxNOW® Test.
Five (5) different operators each interpreted 20-40 devices run at each dilution for a total of 100-200 determinations per dilution. The following results identify a 1:250 dilution of this particular patient urine as the BinaxNOW® Test LOD.

Urine Dilution	Positive Results per Devices Run	Overall Detection
1:200	100/100	100%
**1:250**	**95/100**	**95%**
1:300	160/200	80%
1:400	44/100	44%
1:600	8/100	8%

Clinical sensitivity and specificity (Retrospective Study)

As part of the retrospective study, urine specimens from 35 blood culture positive pneumococcal pneumonia patients and 338 presumed *S. pneumoniae* negative patients (373 total patients) were collected at 3 different facilities and evaluated in the BinaxNOW® Test. BinaxNOW® Test performance was calculated using standard methods. Sensitivity was 86%, specificity was 94%, and overall accuracy was 93%. Ninety-five percent (95%) confidence intervals are listed below.

Sensitivity = 86% (71% - 94%)
Specificity = 94% (91% - 96%)
Accuracy = 93% (90% - 95%)

Clinical sensitivity and specificity (Prospective Study)

In a separate seven-center prospective study, the BinaxNOW® Test was used to evaluate urine specimens collected from 215 hospitalized and outpatients presenting with lower respiratory symptoms or sepsis and from patients otherwise suspected of pneumococcal pneumonia. Patients were considered positive for pneumococcal pneumonia if diagnosed by positive blood culture. The BinaxNOW® Test performed equivalently on both outpatients and hospitalized patients. Ninety-five percent (95%) confidence intervals are listed below.

Outpatient Performance:

Sensitivity = 90% (70% - 97%)
Specificity = 78% (70% - 85%)
Accuracy = 80% (72% - 86%)

Hospitalized Patient Performance:

Sensitivity = 90% (60% - 98%)
Specificity = 71% (59% - 80%)
Accuracy = 73% (62% - 82%)

3) Idaho Technology Inc: FilmArray® Respiratory Panel

Marketed as having POCT potential. A multiplex PCR from nasopharyngeal swabs that identifies 15 respiratory viruses. The test integrates sample preparation, amplification, detection, and analysis. The total hands on time is 2 minutes and the test results are available in 1 hour. No precise measuring or pipetting is required.

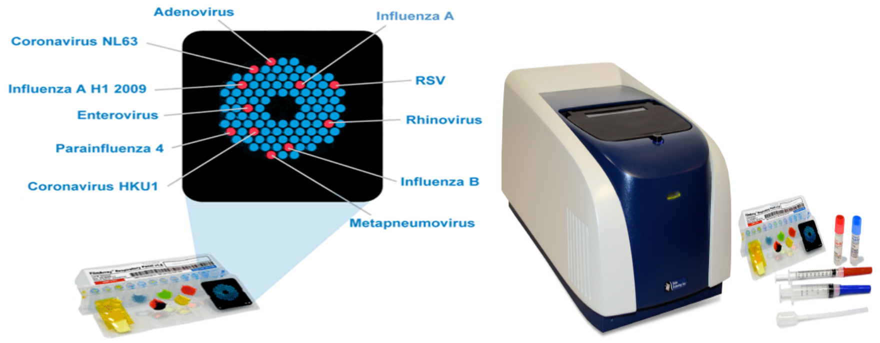

Virus	Sensitivity		Specificity
	Prospective	Retrospective	Prospective
Adenovirus	88.9%	100%	98.3%
Coronavirus HKU1	95.8%	n/a	99.8%
Coronavirus NL63	95.8%	n/a	100%
Human Metapneumovirus	94.6%	n/a	99.2%
Rhinovirus/ Enterovirus	92.7%	95.7%	94.6%
Influenza A	90.0%	100%	99.8%
Influenza A/H1	n/a	100%	100%
Influenza A/H3	n/a	100%	100%
Influenza A/H1-2009	88.9%	100%	99.6%
Influenza B	n/a	100%	100%
Parainfluenza Virus 1	100%	97.1%	99.9%
Parainfluenza Virus 2	87.4%	100%	99.8%
Parainfluenza Virus 3	95.8%	100%	98.8%
Parainfluenza Virus 4	100%	100%	99.9%
Respiratory Syncytial Virus	100%	n/a	89.1%

4) A number of tests to detect respiratory viruses including influenza A and B

(Excel file with information on a number of these tests has been provided separately to RAPP-ID partners)

Examples of Influenza antigen Flu A and B detection tests (CLIA-waived)

Very simple tests, suitable for point of care. Results in 10-15 minutes. Include tests below plus Becton-Dickinson FluA+B, Thermo Electron FLU OIA, ZstatFlu etc

QuickVue Influenza A+B test

Clinical Sample	Sensitivity	Specificity	PPV	NPV
Nasal swab	A-94%, B-70%	A-90%, B-97%	A-62%, B-82%	A-99%, B-94%
**Nasopharyngeal (NP) Swab**	**A-83%, B- 62%**	**A-89%, B- 98%**	**A-67%, B-80%**	**A-95%, B- 95%**
Nasal wash / Nasal aspirate	A-77%, B-82%	A-99%, B-99%	A-91%, B-90%	A-96%, B-97%

BinaxNOW Influenza A&B Test (test performance vs cell culture)

Clinical Sample	Sensitivity	Specificity
NP Wash / Aspirate	A-89%, B-53%	A-95%, B-94%
**Nasopharyngeal Swab**	**A-70%, B- N/A**	**A-90%, B- 98%**
Overall	A-83%, B-53%	A-93%, B-96%

Requirements for Clinical Trial Use (will be produced as a separate document)

The detection of viral pathogens was seen as a requirement for clinical trial use as antiviral agents for common respiratory pathogens other than Influenza A and B are currently in development.
The rapid and specific detection of Gram-negative bacteria for clinical trial use was highlighted in qualitative interviews with clinical trialists.

11.2 Definitions of test outcomes

Condition (determined by "Gold Standard")	
Condition Positive	Condition Negative

**Test Outcome**	**Test Outcome Positive**	**True Positive**	**False Positive** (Type I error)	**Positive Predictive Value=** Number of true positives / Number of true positives + number of false positives
	**Test Outcome Negative**	**False Negative** (Type II error)	**True Negative**	**Negative Predictive Value=** Number of true negatives / Number of true negatives + number of false negatives
		**Sensitivity =** **True Positive / Condition Positive**	**Specificity =** **True Negative / Condition Negative**	

Positive Predictive Value = Sensitivity x Prevalence / (Sensitivity x Prevalence) + (1-Specificity) (1-Prevalence)

Negative Predictive Value = [Specificity x (1-prevalence)] / [Specificity x (1-prevalence)] + [(1-sensitivity) x prevalence]

12. Appendix 2. Definitions of lower respiratory tract infections included in survey

Table 1: Definitions of Lower respiratory tract infections provided in the questionnaire.

CLINICAL CONDITION	CLINICAL DEFINITION
**Community acquired pneumonia (CAP)**	An acute infection of the pulmonary parenchyma that is associated with at least some symptoms of acute infection accompanied by ausculatory findings consistent with pneumonia (such as localized breath sounds and localized rales) and/or the presence of an acute infiltrate on chest radiograph, in a patient not hospitalized or residing in a long-term care facility for 14 days prior to onset of symptoms.
**Influenza**	A person presenting with influenza like illness. Including both seasonal and pandemic influenza.
**Acute exacerbation of asthma**	The acute onset of an increase in asthma symptoms (e.g. dyspnoea, wheeze), a productive cough, and increase and/or change in colour (e.g. green or yellow) of sputum and/or other symptoms suggestive of infection.
**Acute exacerbation of chronic obstructive pulmonary disease (aeCOPD)**	A combination of, or all three of; worsening of dyspnea, increase in sputum purulence and volume, as well as one of the following clinical criteria: an upper respiratory infection in the past five days; fever without another apparent cause; increased wheezing; increased cough; or increase in respiratory rate or heart rate by 20% above the baseline.
**Acute bronchitis**	The acute or sub-acute onset of cough accompanied with evidence of sputum production in a patient with no history of chronic pulmonary disease and no evidence of pneumonia or sinusitis.

13. Appendix 3: Free text comments received from survey

Table 1. Comments on clinicians' priority for the development of a new POCT

CLINICAL CONDITION	NUMBER OF POSITIVE RESPONSES (N) AND EXAMPLES OF COMMENTS	NUMBER OF NEGATIVE RESPONSES (N) AND EXAMPLES OF COMMENTS
**Community acquired pneumonia (CAP)**	(11)<br>*'Differentiating serious pulmonary infection (pneumonia) from other respiratory, mostly self-limiting RTIs is important for management and prognosis. Clinical diagnosis of pneumonia is poor!'*	(9)<br>*'In this case there are sufficient diagnostic signs to base a decision on'*<br><br>*'I would treat with antibiotics anyway'*
***Influenza**	*(8)*<br>*'Identification of influenza would be useful both from epidemiological point of view and for targeting treatment and advice of the patient'*<br><br>*'In a busy flu season it would be helpful to identify patients who might require antibiotic for another pathogen or added infection'*<br><br>*'Influenza is often mixed with influenza-like illness; would be great if we could more objectively separate influenza from other LRTI, as well as following-up the patient during illness period; influenza is also frequently found in pneumonia patients'*<br><br>*'..need diagnostic help for pandemic influenza. Diagnostics for seasonal influenza would be a profoundly inappropriate use of resources'*	*(8)*<br>*'It would not change my management. Antivirals are usually not indicated. Maybe there could be some use in certain risk patients'*<br><br>*'I see no reason to do extra testing in such patient without clinical suspicions of complications'*
**Acute exacerbation of asthma**	*(7)*<br>*'The decision as to whether or not to treat possible associated LRTI in exacerbations of asthma is a recurrent issue in GP. To be able to distinguish between LRTI and viral urticaria would be of great help'*<br><br>*'The risk of further complication from asthma exacerbation could be decreased if we can totally rule out a bacterial aetiology'*	*(5)*<br>*'The treatment of exacerbation of asthma depends on the degree of dyspnea. A POC spirometry could be very useful. I don't know if a POC to detect infection would be useful'*<br><br>*'A negative test could not change my management'*
**Acute exacerbation of chronic obstructive pulmonary disease (aeCOPD)**	(8)<br>*'A POCT could avoid overprescribing antibiotics'*<br><br>*'Useful especially in chronic patients to know when antibiotics might be useful'*<br><br>*'Recurrent antibiotic treatments that may increase the risk of the patient to harbour resistant bacteria. A test that would help target the treatment would be useful'*	*(3)*<br>*'This is pretty much a clinical diagnosis; can't see much benefit in POC for this presentation'*<br><br>*'Would still treat with antibiotics plus steroids as per local protocols'*
**Acute bronchitis**	*(5)*<br>*'Important to differentiate acute bronchitis from pneumonia; clinically hard to differentiate*	*(9)*<br>*'Clinical history and a thorough auscultation can be in most of the cases enough to make the*

	in primary care, but acute bronchitis rarely asks for antibiotic management. However, up to 80% of acute bronchitis will be treated with pneumonia'<br><br>*'A negative test would support me in the management'*	*diagnosis'*<br><br>*'Most of these are pretty straightforward; only need test to distinguish from pneumonia in some cases'*
**Additional clinical priorities for development of a new POCT**	*(4)*<br>*'It may be hard to distinguish bronchitis from atypical pneumonia (Chlamydia p., Mycoplasma p.).*<br><br>*It would be very useful with a POCT to single out atypical pneumonia'*<br><br>*It would be helpful to have a POCT for specific potentially serious infections like Legionella (already existing), Coxiella burnettii infections… SARS, etc.*<br><br>*(6)*<br>*A test that allows differentiation between CAP and bronchitis would be useful.*	
**Additional comments on POCT development**	*(5)*<br>*'POCT potentially useful and could save patients a trip to hospital;*<br><br>*'I think that the place of POCT is at the end of clinical reasoning where uncertainty regarding treatment remains. Treating the results instead of the clinical diagnosis and picture will shift and not diminish the use of antibiotics'*<br><br>*' Apart from the scientific arguments to consider POCT for daily general practice other arguments are becoming more relevant: fear of missing serious illnesses, less (societal) acceptance of false-negative results, despite of enlarging risk of false positive results, service for patients, (double) aging problem, etc'*<br><br>*'A pitfall could be that every POCT positive for bacterie leads to an antibiotic prescription'*<br><br>*'In general, the use of near patient testing would be less useful when a patient has 'barn door' symptoms of illness as it's unlikely that my management would change significantly'*	

Table 2. Comments received in response to technical and operational survey questions

Area of survey	**Examples of representative comments received**<br>(Number of comments)
Treatment decisions to be guided by a new CA-LRTI POCT	(5)<br>'*initial treatment targeting - whether to prescribe an antibiotic or not'* should have been included in options'
Patient age group that a new CA-LRTI should benefit	(2)<br>'ideal test should be available for all age groups'<br>(4)<br>*'in neonates, we are very careful and start antibiotics easily when infection appears'*<br><br>*'if I was considering near patient testing in a child, I would probably be admitting them..'.*
Category of staff that would use the test most often	(8)<br>*'We have no nurses, practice nurse or nurse practitioner'*<br><br>*'Depends on the organisation of the healthcare system'*

Aetiological agents and antibiotic resistances for CA-LRTI POCT detection	(5)<br>*'I think it would depend on local prevalence'*<br><br>*'A test that could highlight an atypical organism may well have more clinical value..'*<br><br>*'Colonisation is important disturbing problem in microbiologic work-up'*
POCT and detection of antibiotic resistance	(3)<br>*'would depend on the country, and this would be directly related to my prescribing pattern'*<br><br>*'.. the ones we use! I haven't heard of half of the above!!'*
Test prediction and speed	(6)<br>*'Bearing in mind an average consultation in primary care is 10 mins.'*<br><br>*'Even shorter time periods, preferably within 3-5 minutes.'*<br><br>*'4 hours or less would be ideal'*<br><br>*'If not instant sub 4 hours is good'*
**Cost of instrumentation and test**	(6)<br>*'Maximum €500'* (for instrument)<br><br>*'even €10 would be too much'* (test)<br><br>*'€1 is the maximum acceptable test cost'*<br><br>*'depends on who is paying'*

Table 3. Clinicians perceived barriers to the uptake of a new POCT in primary care

Area of potential barrier	Examples of comments<br>(Number of comments)
**Time**<br>(time to obtaining the result, to take the test, and in relation to the clinical setting)	(10)<br>*'time constraints of primary care consultations particularly with multimorbidity'*<br><br>*'Time consuming'*<br><br>*'Workload'*
**Cost**<br>(cost of test and instrumentation, and in terms of cost effectiveness)	(19)<br>*'High cost of a POCT'*<br><br>*'Cost/benefit ratio = not convincing'*
**Clinical benefit**	(5)<br>*'..confidence that test would have a significant impact on management of a condition'*<br><br>*'scientific proof of benefit'*<br><br>*'limited added value'*
**Test complexity and ease of use**	(11)<br>*'Complexity of the rapid test'*

	'Difficult to use and/or interpret'<br><br>*'Frequency of use. It has to be quite high, or we will never get used to it'*<br><br>*'Numbers for a complex test to be performed reliably'*<br><br>*'Difficulty of use'*
**Technical requirements of instrumentation and test**	(8)<br>*'Robustness'*<br><br>*' Not having next to patient'*<br><br>*'personnel training'*<br><br>*'quality control'*
**Performance and accuracy**	(7)<br>*'Reliability of the test results'*<br><br>*'Sensitivity / specificity'*
**Clinical samples and clinical pathway considerations**	(3)<br>*'Patient acceptability (if test is invasive)'*<br><br>*'Difficulty of getting sample(s) to practice'*<br><br>*'Sticking children'*
**Other**	(1)<br>*'Generally lack of widespread use of results in internal practice….. peer pressure to stick to traditional methods and abandon innovation'*

14. References

1. **Hobbs R** *et al*. Near patient testing in primary care. ***BMJ*** 1996;312:263-264

2.**Delaney BC**, *et al* . Systematic review of near patient test evaluations in primary care. ***BMJ*** 1999; 319:824-827.

3. **Waterer GW** *et al*. Management of community-acquired pneumonia in adults. ***Am J Respir Crit Care Med*** 2011; 183:157-164.

4. **Lim WS** *et al*. British Thoracic Society adult community acquired pneumonia audit 2009/2010. ***Thorax*** 2011; 66:548-9.

5. Woodhead M *et al*. Community-acquired pneumonia on the intensive care unit: secondary analysis of 17,869 cases in the ICNARC case mix programme database. ***Crit Care*** 2006; 10 Suppl 2:S1.

6. **BTS guidelines** for the management of community acquired pneumonia in adults: update 2009. **Thorax** 2009; *64(Supp III).*

7 **Bartlett J.G.**. Diagnostic tests for agents of community-acquired pneumonia ***CID*** 2011:52 (Suppl 4); 296-304

8 **Jones RN** *et al*. Evolving trends in *Streptococcus pneumoniae* resistance: implications for therapy of community-acquired bacterial pneumonia. ***Int J Antimicrob Agents*** 2010; 36: 197–204

9. **Dicpinigaitis PV** *et al*. Acute cough: a diagnostic and therapeutic challenge. ***Cough*** 2009, 5:11 doi:10.1186/1745-9974-5-11

10. **Cough Guideline***.* Recommendations for the Management of Cough in Adults· ***Thorax*** 2006, 61 (Suppl 1)i1-i24

11. **Lindbaek M.** Prescribing antibiotics to patients with acute cough and otitis media. ***Br J Gen Pract***. 2006 Mar; 56(524):164-6

12**. Gritzfeld JF** *et al*. Comparison between nasopharyngeal swab and nasal wash, using culture and PCR, in the detection of potential respiratory pathogens.***BMC Research Notes*** 2011 4:122

13**. Van Heirstraeten** *et al*. Quantitative comparison of *Streptococcus pneumoniae* in patients with community acquired lower respiratory tract infections and in matched healthy controls. Poster presentation D-170 ***ICAAC*** 2010. https://www.grace-lrti.org/... /20100819 poster ICAAC colonization final

14. **Vernet G** *et al*. Laboratory-based diagnosis of pneumococcal pneumonia: state of the art and unmet needs. ***Clinical Microbiology and Infection*** 10.111/j.I469-0691.2011.03496.

15. **Bartlett J.G.** Diagnostic tests for agents of community-acquired pneumonia ***CID*** 2011:52 (Suppl 4); 296-304

16. **Johansson N** et al. 2010. Aetiology of community-acquired pneumonia: increased microbiological yield with new diagnostic methods. **CID** 2010:50 (January); 202-209

17. **Zakharkina T** *et al* Detection of microorganisms in exhaled breath condensate during acute exacerbations of COPD. ***Respirology*** 2011;16:932-938

18**. Besa V** *et al.* Volatile organic compounds (VOCs) in COPD patients with exacerbation. Poster P4788 presented at 2011 ***European Respiratory Society*** Conference, Amsterdam

19. **Ishimaru M** *et al*. Analysis of volatile metabolites from cultured bacteria by gas chromatography/atmospheric pressure chemical ionization–mass spectrometry. ***J Breath Res****.* 2008. 2 037021 doi:10.1088/1752-7155/2/3/037021

20**. Randall A** et al Detection of volatile metabolites produced by bacterial growth in blood culture media by selected ion flow tube mass spectrometry. ***J Microbiol Methods*** 2006. 65: 361-365

21. **Holm A** *et a*l Aetiology and prediction of pneumonia in lower respiratory tract infection in primary care. ***BJGP***. 2007; 57:547-554

22. **Van Eldere** LJR *et al* Clinical diagnosis of influenza virus infection: evaluation of diagnostic tools in general practice. ***BJGP*** 2001;51:630-634.

23. **Lalkhen AG et al.** Clinical tests: sensitivity and specificity. ***Continuing Education in Anaesthesia, Critical Care and Pain***. 2008; 8(6):221-223
